# Serial entrepreneurs and the macroeconomy

**DOI:** 10.1007/s11187-026-01213-8

**Published:** 2026-04-22

**Authors:** Micole De Vera, Sónia Félix, Sudipto Karmakar, Petr Sedláček

**Affiliations:** 1 Bank of Spain and IZA, Madrid, Spain; 2 Bank of Portugal and Nova School of Business and Economics, Lisbon, Portugal; 3 Bank of England and King’s College London, London, England; 4 University of New South Wales, Sydney, 2052 NSW Australia

**Keywords:** Serial entrepreneurs, Business dynamism, Macroeconomy, Job creation, D22, E24, L1

## Abstract

Entrepreneurship is crucial for job creation and economic growth. But not all entrepreneurs are alike. For instance, firms of serial entrepreneurs — owners of multiple businesses — have been documented to outperform other businesses. In this paper, we provide novel evidence showing that firms of serial entrepreneurs are also important for macroeconomic outcomes. Using unique administrative data from Portugal, we document that serial entrepreneur firms (i) disproportionately contribute to aggregate job creation and productivity growth, (ii) help shape aggregate business dynamism, and (iii) are more likely to be high-growth (“gazelles”). This superior performance is largely driven by entrepreneurs’ education, ability, past managerial experience, and lower indebtedness of their firms. We discuss the implications of our findings for macroeconomic models — which typically ignore serial entrepreneurs.

**Supplementary Information:**

The online version contains supplementary material available at https://doi.org/10.1007/s11187-026-01213-8.

## Abstract (plain language summary)

While only 4% of entrepreneurs own multiple businesses at once, their firms play a disproportionately important role for the macroeconomy. Drawing on unique administrative data, we find that these firms (i) contribute substantially to overall job creation and productivity growth, (ii) influence broader patterns of business dynamism, and (iii) are more likely to be high-growth “gazelles.” These insights underscore the importance of examining serial entrepreneurship through a macroeconomic lens — a perspective that remains underexplored both empirically and theoretically.

**Supplementary Information:**

The online version contains supplementary material available at https://doi.org/10.1007/s11187-026-01213-8.

## Introduction

Policy makers have long recognized the important role entrepreneurship plays in job creation and economic growth (see, e.g., Mata and Portugal, 2004; Cagetti and De Nardi, 2006). However, entrepreneurs and their firms are not all alike. Indeed, vast heterogeneity exists in terms of individual characteristics of business owners and the various dimensions of performance of their firms (see, e.g., Westhead and Wright, 1998; Haltiwanger et al. 2013). Therefore, policy makers may need to put in place targeted policies supporting varying needs of different types of entrepreneurs in order to exploit their full potential (see, e.g., OECD 2020).

An important dimension of heterogeneity among entrepreneurs is the number of firms they own. Indeed, existing research documents that firms of “serial entrepreneurs” — business owners with multiple firms — tend to outperform other businesses (see, e.g., Shaw and Sørensen, 2019; Carbonara et al., 2020). Against this backdrop, and especially in the current environment in which many countries face lackluster aggregate growth, policy makers may strive to identify high-growth firms, including those of serial entrepreneurs, and consider (re-)allocating resources towards them. However, to the best of our knowledge, there is no established evidence that firms of serial entrepreneurs are, in fact, important for aggregate outcomes. While individual firms of serial entrepreneurs outperform other businesses, it does not immediately follow that — as a group — serial entrepreneur firms are powerful enough to leave a footprint on the macroeconomy. Our paper fills this gap.

In particular, we make use of unique administrative data covering the near universe of workers, firms, and business owners in Portugal — the “Quadros de Pessoal” (QP). With this data — which spans 1986–2017 — we show that firms of serial entrepreneurs (i) disproportionately contribute to aggregate job creation and productivity growth, (ii) help shape aggregate business dynamism, and (iii) are more likely to be high-growth businesses (so-called “gazelles”). Moreover, we show that the documented superior performance of serial entrepreneur firms can largely be traced back to their lower indebtedness and the education, ability, and past managerial experience of their founders. Therefore, our paper provides novel evidence that serial entrepreneurs are, in fact, important drivers of macroeconomic outcomes and points towards possible avenues for future research into targeted policies for supporting entrepreneurship.

There are many ways in which to classify entrepre-neurs. To streamline our analysis, we focus only on two groups: “portfolio serial entrepreneurs” and “other” entrepreneurs. Portfolio serial entrepreneurs are defined as business owners who simultaneously own at least two firms at some point within our sample.^1^ Intuitively, all remaining business owners fall into the category of “other” entrepreneurs. We then categorize firms accordingly: portfolio serial entrepreneur (SE-P) firms are owned by portfolio serial entrepreneurs, while other (O) firms are all other businesses. At first glance, portfolio serial entrepreneurs and their businesses may not inspire great interest from a macroeconomic perspective. Only about 3.5% of all business owners are portfolio serial entrepreneurs, and their firms represent 13.5% of all businesses. However, we document that SE-P firms are responsible for about 20% of *aggregate* productivity growth and almost 25% of *all* job creation and employment.

Next, we turn to analyzing the impact of SE-P firms on overall business dynamism. As other economies, Portugal is also characterized by so-called “up-or-out” dynamics by which young firms face high rates of exit, but surviving young businesses display strong growth. Our results show that, compared to other businesses, SE-P firms enter larger and grow faster throughout their life-cycle. In contrast, other firms are characterized by higher exit rates. This job creation prowess of SE-P firms is related to the fact that, compared to other businesses, they are about three times more likely to be high-growth firms, so-called “gazelles.”^2^

The last part of our analysis is devoted to investigating the possible sources of such differences. We focus on two broad categories: “learning” (see, for example, Cope, 2005; Politis, 2005; Lazear, 2005; Eggers and Song, 2015; Lafontaine and Shaw, 2016) and “selection” (see, for example, Ucbasaran et al., 2008; Sedláček and Sterk, 2017; Shaw and Sørensen, 2022; Sanguineti et al., 2025). We interpret both terms broadly. In particular, while “learning” may explicitly include improvements in the skills of business owners, it may also cover other ex-post or dynamic forces such as the gradual accumulation of net worth. In contrast, “selection” focuses on the various, ex-ante, factors with which serial entrepreneurs (and their firms) may be endowed.

Our data offers a natural way of isolating the impact of learning and ex-post factors on firm performance. In particular, we do so by separately analyzing the performance of “first” (FSE) and “subsequent” (SSE) businesses of portfolio serial entrepreneurs. Intuitively, FSE firms are those businesses which entrepreneurs owned *before* they could be classified as portfolio serial entrepreneurs. In contrast, SSE businesses are the cause of the portfolio serial entrepreneur classification and constitute their second and following firms.

Under the presumption of learning and (favorable) ex-post forces, subsequent firms of portfolio serial owners should outperform their “first” business. Indeed, our results suggest that learning plays a role in explaining some of the estimated differences. In particular, compared to FSE firms, SSE businesses are about a quarter larger.^3^ That said, learning does not seem to be the only driver of success. In fact, FSE businesses already considerably outperform O firms, suggesting that selection also plays a role.

Therefore, we consider a range of, ex-ante, aspects on which portfolio serial entrepreneurs (and their firms) may be selecting — entrepreneur characteristics, employee characteristics, and financial factors. To incorporate the latter, we merge our baseline QP data with the “Sistema de Contas Integradas das Empresas” (SCIE), which contains balance sheet and income statement information. Equipped with this unique merged dataset, we estimate the extent to which differences in performance between SE-P and O firms — so-called “serial entrepreneur premia” — can be accounted for by our explanatory factors. Two findings stand out.

First, entrepreneur characteristics alone can account for between about 20 and almost 50% of the estimated premia. Within these, the most important factors are entrepreneur education, past wages — a proxy of entrepreneurs’ ability — and past managerial experience.^4^ Interestingly, whether or not business owners are also managers seems to matter less for the estimated premia.

Next, financial factors are the second most important determinant of the superior performance of SE-P firms. In particular, lower indebtedness of SE-P firms alone can explain between 20 and 50% of the exit and growth premia. Moreover, financial factors remain to explain some of the premia even when we restrict our analysis only to *first* firms of portfolio serial entrepreneurs. This suggests that portfolio serial business owners enter entrepreneurship with a healthier balance sheet to begin with. Quantitatively, however, the contributions of financial factors to the estimated premia are stronger among subsequent firms of portfolio serial entrepreneurs. This suggests that the initial success of the first businesses of portfolio serial entrepreneurs helps make the financial position of their subsequent firms even more comfortable, allowing them to thrive further.

The remainder of the paper is structured as follows. The next section provides a literature review. Next, we describe our data, definitions, and document basic descriptive statistics. Section 4 reports results pertaining to the impact of portfolio serial entrepreneurs on the macroeconomy and on business dynamism in particular. Section 5 analyzes potential sources of the superior performance of SE-P firms. The last two sections provide a discussion and concluding remarks.

**Table 1:** Categorization of entrepreneurs by number of firms owned

I. Number of firms owned: one	
*Novice entrepreneurs*	
see, e.g., Scott and Rosa (1996); Westhead and Wright (1998); Shaw and Sørensen (2019)	
II. Number of firms owned: multiple	
*Multiple business owners*, see, e.g., Scott and Rosa (1996)	
*Habitual entrepreneurs*, see, e.g., Westhead and Wright (1998)	
*Serial entrepreneurs*, see, e.g., Shaw and Sørensen (2019)	
$$\overbrace{\phantom{0000000000000000000000000000000000000000000000000000000000000000000000000000000000000000000000}}$$	
II.a. Multiple firms simultaneously	II.b. Multiple firms sequentially
*Portfolio entrepreneurs*	*Sequential entrepreneurs*
see, e.g., Scott and Rosa (1996);	see, e.g., Shaw and Sørensen (2019)
Westhead and Wright (1998);	*Serial entrepreneurs*
Shaw and Sørensen (2019)	see, e.g., Westhead and Wright (1998)

## Literature review

There is a vast literature studying entrepreneurship — both in terms of an occupational choice contrasting paid employment and in terms of its macroeconomic impacts. Relatively less is known about the determinants of serial entrepreneurship and, to the best of our knowledge, we are the first to analyze the macroeconomic footprint of serial entrepreneurs.

### Entrepreneurship vs employment

Early contributions to the entrepreneurship literature include studies analyzing the occupational choice between entrepreneurship and paid employment. One strand of research emphasizes the psychological, sociological, and cognitive aspects, e.g., individual differences in risk aversion or “entrepreneurial skills” and institutional factors (see, e.g., Lucas, 1978; Lazear, 2005; Carbonara et al. 2017). Another strand of the literature emphasizes how differences in earnings (and their volatility) between entrepreneurship and employment interact with individuals’ wealth and financial position (see, e.g., Evans and Jovanovic, 1989; Cagetti and De Nardi, 2006). Moreover, despite the fact that average pecuniary returns may not be higher in entrepreneurship, some individuals may still prefer it based on non-pecuniary factors such as autonomy and flexibility (see, e.g., Hamilton, 2000; Benz and Frey, 2008)

### Serial entrepreneurship

Table 1 visualizes how existing studies classify entrepre-neurs on the basis of the number of businesses they own. In particular, entrepreneurs who own a single business are relatively consistently referred to as “novice” entrepreneurs.^5^ In contrast, there is less consensus about the terminology of entrepreneurs who own multiple businesses. Such business owners have been referred to as “serial entrepreneurs”, “habitual entrepreneurs,” and “multiple business owners” (see, e.g., Scott and Rosa, 1996; Westhead and Wright, 1998; Shaw and Sørensen, 2019).

In addition, entrepreneurs who own multiple businesses are further classified based on whether they do so simultaneously or sequentially. Typically, entrepreneurs who own multiple businesses at the same time are referred to as “portfolio entrepreneurs” (see, e.g., Westhead and Wright, 1998; Shaw and Sørensen, 2019). In contrast, the terminology referring to entrepreneurs who own multiple businesses but who do so sequentially (such that they own only a single business at a time) is somewhat mixed. These business owners have been referred to as “sequential entrepreneurs” (see, e.g., Shaw and Sørensen, 2019) and “serial entrepreneurs” (see, e.g., Westhead and Wright, 1998).

Finally, the literature also makes a distinction between serial entrepreneurship involving start-ups and that involving existing firms (see, e.g., Wright et al., 1997; Ucbasaran et al., 2008). In the former case, serial entrepreneurs are business founders. In the latter case, serial entrepreneurs are investors in existing businesses.

These classifications are substantive, as existing research has identified important differences between such groups of entrepreneurs. For example, evidence points to serial entrepreneurs possessing greater prior entrepreneurial and managerial experience (see, e.g., Westhead and Wright, 1998), enjoying higher incomes and sales (see, e.g., Chen, 2013; Lafontaine and Shaw, 2016) and remaining in business for longer (see, e.g., Shaw and Sørensen, 2019). Similarly, a distinction between owner-managers and owner-investors is particularly important when analyzing firm performance because these two groups of entrepreneurs have different incentives and are differently involved in actively running the business (see, e.g., Fahlenbrach, 2009)

Throughout this paper, we adopt the terminology of Shaw and Sørensen (2019) and distinguish between novice, portfolio, and sequential entrepreneurs, with the latter two groups referred to as serial entrepreneurs. The next section describes how exactly we identify these groups in our data and how we deal with owners-founders and owners-investors.

### Determinants of (serial) entrepreneurship

Existing research broadly identifies the following drivers of (serial) entrepreneurship: human capital, learning, and selection mechanisms.

Education and prior entrepreneurial experience have been identified as allowing for better opportunity recognition and exploitation (see, e.g., Ucbasaran et al., 2008; Choi et al., 2021; Queiró, 2022). Serial entrepreneurs accumulate what has been termed “entrepreneurial capital,” which may increase the likelihood of subsequent venture creation (see, e.g., Westhead et al., 2005).

Other studies emphasize the importance of selection mechanisms with higher-ability individuals or those with greater financial means being more likely both to become entrepreneurs and to re-enter entrepreneurship (see, e.g., Parker, 2013; Carbonara et al., 2020; Brandt et al., 2025). However, the extent to which observed patterns reflect learning versus selection on innate ability remains challenging to determine. Moreover, other factors which are harder to observe (or indeed are unobserved) — such as the institutional setting, depth of venture capital markets, size of professional networks and other “social capital” — are also likely influential (see, e.g., Ucbasaran et al., 2003; Wright et al., 1998).

### Entrepreneurship and the macroeconomy

From a macroeconomic perspective, entrepreneurship has predominantly been studied as a channel influencing (top) income inequality (see, e.g., Cagetti and De Nardi, 2006; Gabaix et al., 2016; Piketty et al., 2018). While firm heterogeneity has been identified as key for understanding aggregate outcomes (see, e.g., Decker et al., 2020; Sedláček and Sterk, 2017), macroeconomic models incorporating business dynamism typically abstract from the occupational choice between paid work and entrepreneurship (see, e.g., Hopenhayn and Rogerson, 1993; Clementi and Palazzo, 2016) with notable exceptions including, e.g., Jiang and Sohail (2023) and Kozeniauskas (2025).^6^

To the best of our knowledge, from a macroeconomic perspective, serial entrepreneurship is not well understood — neither theoretically nor empirically. This paper fills the gap and documents that businesses of serial entrepreneurs are indeed important for aggregate outcomes.

## Data, definitions, and basic descriptive statistics

The main purpose of this paper is to document the contribution of serial entrepreneurs — and their businesses — to macroeconomic outcomes. Towards this end, we begin by describing our primary data source and laying out the definitions of key variables. We pay particular attention to the classification of entrepreneurs, which is central to our analysis.

Before presenting our results in the next sections, we show basic descriptive statistics concerning business dynamism and serial entrepreneurship. In what follows, we use the terms entrepreneur and (business) owner interchangeably.

### Data

Our main data source is Quadros de Pessoal (QP), a Portuguese census of private sector employees conducted annually by the Ministry of Employment, Solidarity and Social Security (MESSS). The QP is a rich administrative employer-employee matched panel dataset which, for our purposes, possesses a unique advantage over other similar datasets — it contains information on business *owners*.^7^ Since the QP has been extensively used in the literature (see, e.g., Mata and Portugal, 2004; Carneiro et al., 2012; Queiró, 2022), we defer a general description of the data to the Appendix. Instead, in what follows, we only focus on key aspects directly related to our analysis.

The QP covers the universe of firms between 1986 and 2017 with at least one employee. Firms represent our main units of analysis and, unless explicitly stated otherwise, we use the terms firm and business interchangeably. Thanks to the longitudinal linkages inherent to the QP, we are able to connect firm-level characteristics with an individual business owner and track owners, their businesses, and the workers within these businesses over time.

For some of our analyses, we merge our QP data with information in Sistema de Contas Integradas das Empresas (SCIE). The latter contains firm-level information on balance sheets and income statements. To preserve the coverage and definition of variables, we focus on the years 2010–2017 when using the SCIE. In the Appendix, we show that our baseline results are robust to considering different sub-periods.

### Definitions

This subsection defines key variables of interest. We begin by discussing our categorization of entrepreneurs and how it relates to existing studies of entrepreneurship.

#### Types of entrepreneurs and their firms

As we have discussed in the previous section, we adopt the classification of entrepreneurs from Shaw and Sørensen (2019). In particular, we distinguish between the following groups of business owners:

1. *Novice entrepreneurs*: ever own only a single business at some point in our sample,
2. *Serial entrepreneurs*: own multiple businesses at some point in our sample,
  1. *Portfolio serial entrepreneurs*: own multiple businesses simultaneously at some point in our sample,
  2. *Sequential serial entrepreneurs*: own multiple businesses at some point in our sample, but never more than one at a time.

While our data does not allow us to determine the motivation behind business ownership, we can approximate the distinction between owner-managers (founders) and owner-investors by making use of the QP’s information on job hierarchies. Specifically, we classify business owners as managers if they also fall into the top level of hierarchy: “top executives (top management).”^8^ According to this definition, 98 percent of serial entrepreneurs are in fact classified as owner-managers in our sample and, therefore, we do not explicitly consider this distinction for the majority of our analysis.

More concretely, in our data, we identify serial entrepreneurs as individuals who — at any given point in our sample — own *some fraction* of more than one firm. Under this classification, serial entrepreneurship is viewed as a “permanent characteristic” (fixed effect).^9^ This gives rise to the following grouping of firms, which inherits the terminology from the classification of business owners:

1. Novice (N) firms: all owners are novice entrepreneurs,
2. Serial entrepreneur (SE) firms: at least one owner is a serial entrepreneur,^10^
  1. Portfolio serial entrepreneur (SE-P) firms: at least one owner is a portfolio serial entrepreneur,
  2. Sequential serial entrepreneur (SE-S) firms: all owners are sequential serial entrepreneurs.

As will become clear below, the performance of SE-S and N firms is similar in our sample, while SE-P firms stand out. Therefore, in order to streamline our analysis, we choose to group together SE-S and N firms into one category — “Other” (O) firms — and compare these to SE-P firms.

#### Outcome variables

To describe the performance of a group of firms, we focus on four distinct variables: size, growth, productivity, and exit rates. In particular, we focus on employment, *E*, as our baseline measure of firm size. Next, we follow Davis et al. (1996), henceforth DHS, and measure firm growth in firm *i* and period *t*, $g_{it}$, as

1 $$\begin{aligned} g_{it}=\frac{(E_{it}-E_{it-1})}{X_{it}}, \end{aligned}$$

where $X_{it}=1/2(E_{it}+E_{it-1})$. Davis et al. (1996) propose this growth rate definition to mitigate potential bias from measurement error and mean regression. Accordingly, job creation (*JCr*) and destruction rates (*JDr*) of a particular group of firms, *s*, can be defined as:

2 $$\begin{aligned} JCr_{t}=\sum _{s}\bigg (\frac{X_{st}}{X_t}\sum _{i\in s, g_{i,t}\ge 0}\bigg (\frac{X_{it}}{X_{st}}\bigg )g_{it}\bigg ), \end{aligned}$$

3 $$\begin{aligned} JDr_{t}=\sum _{s}\bigg (\frac{X_{st}}{X_t}\sum _{i\in s, g_{i,t}<0}\bigg (\frac{X_{it}}{X_{st}}\bigg )g_{it}\bigg ). \end{aligned}$$

Since accurate estimates of firm-level productivity are hard to obtain, we focus on the simplest measure of labor productivity $q_{i,t}=Z_{i,t}/E_{i,t}$, where $Z_{i,t}$ are sales of firm *i* in period *t*.

Average exit rates of a group of firms, *s*, are defined as:

4 $$\begin{aligned} D_{s,t}=\frac{(\#\,\text {of exiting firms})_{s,t}}{(\#\,\text {of firms})_{s,t}}. \end{aligned}$$

Finally, part of our analysis focuses on high-growth firms, so-called gazelles. We follow the Eurostat-OECD (see European Commission, 2007) definition of gazelles as businesses up to 5 years old, with a minimum of 10 employees (at some point in the firm’s existence), and with average annualized growth of at least 20% per year, over a three-year period. As with the definition of serial ownership, we treat the term gazelle as a permanent characteristic — a fixed effect — of a particular business.^11^

**Table 2:** Sectoral composition of serial entrepreneur firms

		Serial entrepreneur firms	
	All firms	Portfolio	Sequential
Wholesale and retail trade	33.0	33.8	33.0
Manufacturing	16.8	15.6	18.3
Construction	13.7	11.4	14.7
Accommodation and food services	11.6	8.7	12.5
Real estate and other activities	11.3	17.6	10.2

Notes: the columns show, respectively, “all,” “portfolio serial,” and “sequential serial” entrepreneur businesses. The values report the shares (in %) of each group of businesses across five broad industries in which almost 90% of all firms operate

### Basic descriptive statistics

Before moving to our central results, we first provide basic descriptive statistics of firm dynamics and serial entrepreneurship in the Portuguese economy.

#### Average firm life-cycle dynamics

Every year on average in the Portuguese economy, about 10% of the youngest firms shut down. This fraction drops to about 8% by the time businesses reach the age of 6 years. At the same time, surviving startups almost double in size in the same time period of 6 years. These “up-or-out” patterns are consistent with those found in the U.S. (see Haltiwanger et al., 2013) and other developed economies (see, for example, Calvino et al., 2015; Sternad and Mödritscher, 2022).

#### Gazelles in the Portuguese economy

Under the Eurostat-OECD definition of high-growth firms, about 9% of all businesses are gazelles in Portugal. That said, given their superior growth performance, this group of firms alone accounts for almost a third of aggregate employment and almost half of all job creation. These patterns are broadly consistent with those in the U.S. economy see, (Haltiwanger et al., 2016).

#### Prevalence of serial entrepreneurship

According to our definitions, we find that 12.7% of all business owners can be classified as serial entrepreneurs. Of these, almost 3/4 of serial entrepreneurs run their businesses sequentially. In contrast, portfolio serial entrepreneurs — the primary focus of this paper — account for only 3.5% of all business owners.

These numbers are in line with Shaw and Sørensen (2019) who — using the near universe of Danish firms and owners — report that about 10% of business owners can be classified as serial entrepreneurs (either portfolio or sequential). Other studies place the fraction of serial entrepreneurs somewhere between one-fifth and one-third of all business owners (see, e.g., Westhead and Wright, 1998; Parker, 2013).

Despite the fact that portfolio serial entrepreneurs account for less than 4% of business owners in Portugal, their firms represent about 13.5% of all businesses. Moreover, serial entrepreneurship appears to be widespread throughout the entire economy, with the sectoral composition of SE businesses closely matching that of the economy as a whole, see Table 2.^12^

**Table 3:** Characteristics of entrepreneurs

		Serial entrepreneurs	
	Novice	Portfolio	Sequential
Age (years)	40.1	41.3	40.4
Education (years)	9.3	10.6	9.0
Female proportion (%)	31.8	23.7	28.7
Wages (log hourly rate)	0.25	0.48	0.20
Manager experience (%)	19.5	52.1	19.6

Notes: the table reports average values across novice and serial (portfolio and sequential) entrepreneurs. While “age,” “education,” and “female proportion” refer to average age, years of schooling and the fraction of female entrepreneurs in the two groups of business owners, “wages” refer to income earned in the last employment spell prior to entrepreneurship and “manager experience” reports the share of entrepreneurs classified as managers in their last employment spell

**Table 4:** Firm characteristics and serial entrepreneur premia

				Premia	
	N	SE-P	SE-S	SE-P vs N	SE-S vs N
Size (workers)	5.3	10.9	5.8	$$0.53^{***}$$	$$0.19^{***}$$
Exit (in %)	7.9	5.4	8.2	$$-1.73^{***}$$	$$0.64^{***}$$
Growth (in %)	8.9	9.8	8.8	$$1.71^{***}$$	$$-0.35^{*}$$
Productivity (all = 1)	0.79	1.17	0.88	$$0.23^{***}$$	0.00

Notes: the columns show, respectively, the unconditional averages of novice and serial (portfolio and sequential) entrepreneur firms and the SE-P premium estimated from regression Eq. 5. The rows depict, respectively, average (employment) size, exit rates, (employment-weighted) net employment growth, and average labor productivity scaled by labor productivity of all firms. Ordinary Least Squares estimates with robust standard errors clustered at the firm level in parentheses. *** stands for statistical significance at the 1% level and * stands for statistical significance at the 10% level

#### Characteristics of serial entrepreneurs

Table 3 shows average characteristics of novice, portfolio, and sequential serial entrepreneurs. The values in the first two columns reveal that — compared to novice entrepreneurs — portfolio serial entrepreneurs are somewhat older, more educated, more often men, earned considerably higher wages, and were more often managers in their last employment spells. Shaw and Sørensen (2019) find similar results in Denmark, where — compared to other business owners — serial entrepreneurs are slightly more educated and more often men. We return to this point in Sect. 5 when investigating the drivers of serial entrepreneurship. The last column shows that — as we eluded to earlier — in terms of business owner characteristics, sequential serial entrepreneurs are closer to novice business owners than to portfolio entrepreneurs.

#### The serial entrepreneur premium

Finally, before investigating the *macroeconomic* impact of serial entrepre-neurs and their businesses, we estimate firm-level differences in various measures of performance between SE and N businesses according to:

5 $$\begin{aligned} y_{i,t} = \alpha + \beta 1\!\!1_{i\in SE} + \gamma F_{i,t} + \epsilon _{i,t}, \end{aligned}$$

where $y_{i,t}$ is an outcome variable of interest, $1\!\!1_{i\in SE}$ is an indicator function which is equal to one if business *i* is a serial entrepreneur firm and zero otherwise. We dub the coefficient β as the average “serial entrepreneur premium” in regards to variable *y*.^13^ In addition, we also include a range of control variables, $F_{i,t}$: firm age, industry, and year fixed effects.

While the first three columns of Table 4 show the unconditional averages of the variables of interest for our two groups of entrepreneurs, the last two columns report the estimated serial (portfolio and sequential) entrepreneur premia, β. The results show that, even conditional on other control variables, SE-P firms are almost 50% larger, their exit rates are about 20% lower, they grow at a 20% faster pace, and they are 23% more productive compared to novice businesses.^14^ These patterns are consistent with previous studies which analyze different countries or industries and typically focus only one or two of the dimensions we examine (see, for example, Chen, 2013; Lafontaine and Shaw, 2016; Shaw and Sørensen, 2019).^15^

In contrast, the last column suggests that there is no such systematic pattern for sequential serial entrepreneurs. While their businesses seem to be larger than those of novice owners, exit rates (growth rates) are higher (lower), and there is no difference in productivity levels. As foreshadowed earlier, in what follows, we will therefore group together firms of novice and sequential serial entrepreneurs into one category: “other” firms.

## Macroeconomic impact of serial entrepreneurs

This section documents the importance of serial entrepre-neurs for *macroeconomic* outcomes, which — to the best of our knowledge — has not been put forward empirically or theoretically. Three key messages emerge out of our analysis. First, SE-P firms are important for aggregate job creation and productivity growth. Second, SE-P businesses help shape aggregate business dynamism. Finally, a key reason behind the previous two findings is that SE-P firms are disproportionately represented among high-growth firms.

**Table 5:** Aggregate productivity growth decomposition

	Total		Within	Between	Cross	Entry	Exit
Aggregate	7.1		13.8	3.0	-8.5	-2.4	1.2
SE-P firms: level	1.4		3.2	0.4	-2.0	-0.2	0.0
SE-P firms: share of aggregate	19.7		23.2	13.3	23.5	8.3	0.0

Notes: the table reports values (in %) from the productivity growth decomposition in Eq. 7. The first row reports aggregates, the second and third rows report the contribution of portfolio serial entrepreneur (SE-P) firms only in levels and as a share of the aggregate, respectively

**Table 6:** Contributions to aggregates (in %): SE-P and other firms

	Firms	Employment	Job creation	Job destruction
Other firms	86.5	76.6	77.3	81.6
SE-P firms	13.5	23.4	22.7	18.4

Notes: the table shows, respectively, the contributions (in %) of “other” and “portfolio serial entrepreneur” (SE-P) businesses to the aggregate number of firms, employment, job creation, and job destruction

### Aggregate productivity growth and job creation

Economists have long strived to identify groups of firms which are most important for driving aggregate outcomes (see, for example, Birch, 1981; Guzman and Stern, 2015; Haltiwanger et al., 2016). It is in this context that we present one of our headline results that firms of serial entrepreneurs contribute disproportionately to aggregate productivity growth and job creation.

#### Contributions to aggregate productivity growth

First, we turn our attention to the contribution of serial entrepreneur firms to *aggregate* productivity growth. Towards this end, we follow Foster et al. (2001) and define industry-specific productivity by

6 $$\begin{aligned} Q_{jt}=\sum _s\sum _{i\in s}\omega _{it}q_{it} \end{aligned}$$

where $Q_{jt}$ is the productivity index of industry *j* in year *t*, *s* is a subset of all businesses (in our case portfolio serial entrepreneur and other firms, i.e., $s=\{SE-P, O\}$), $\omega _{it}$ is the employment share of firm *i* in industry *j* (shares $\omega _{it}\ge 0$ sum to one), and $q_{it}$ is the logarithm of labor productivity at the firm level. Denoting the set of continuing, new, and exiting firms with *C*, *N*, and *X*, respectively, we decompose the change in industry-level productivity as

7 $$\begin{aligned} \Delta Q_{jt}=\sum_{s}\left[\begin{array}{c} \overbrace{\sum_{i \in C_{s}} \omega_{i, t-1} \Delta q_{i t}}^{\mathrm{within}}+\overbrace{\sum_{i \in C_{s}}\left(q_{i, t-1}-Q_{j, t-1}\right) \Delta \omega_{i t}}^{\mathrm{between}}+\overbrace{\sum_{i \in C_{s}} \Delta q_{i t} \Delta \omega_{i t}}^{\mathrm{cross}} \\ +\underbrace{\sum_{i \in N_{s}} \omega_{i, t}\left(q_{i, t}-Q_{j, t-1}\right)}_{\mathrm{entry}}-\underbrace{\sum_{i \in X_{s}} \omega_{i, t-1}\left(q_{i, t-1}-Q_{j, t-1}\right)}_{\mathrm{exit}} \end{array}\right]. \end{aligned}$$

where we follow Foster et al. (2001) and Baily et al. (1992) and compute the above decomposition for every industry-year pair in our data and aggregate up to the entire economy using gross output weights.

The first, “within,” term measures the contributions of productivity changes at the firm-level for a given mix of businesses. The “between” term reflects contributions stemming from a reallocation of market shares from relatively less to relatively more productive businesses. The third, “cross,” term encompasses the combination of the previous two — a reallocation of market shares towards businesses which display increases in firm-level productivity. The last two terms reflect, respectively, the contributions driven by the entry of firms with above-average productivity and the exit of businesses with below-average productivity.

The first row of Table 5 reports average aggregate productivity growth over our sample period and the contributions of the separate components from the decomposition in Eq. 7. The second and third rows show the contributions of portfolio serial entrepreneurs in levels and as a share of the respective components of aggregate productivity growth.

Consistent with other studies (see, for example, Dias and Marques, 2021; Reis, 2013), our decomposition reveals that aggregate productivity growth is predominantly driven by within-firm growth. Importantly for the focus of our paper, the results suggest that SE-P firms are important for aggregate productivity growth. In particular, despite accounting for less than 14% of firms, they alone are responsible for over 23% of the within component of aggregate productivity growth.

Next, note that the contribution of firm entry to aggregate productivity growth is negative overall (top row, second-to-last column). This indicates that in our sample, startups are somewhat less productive than incumbent firms. While this holds true also for startups of serial entrepreneurs, it does so to a lesser extent — consistent with the estimated serial entrepreneur premia.

Finally, relatively less productive firms tend to shut down overall, which contributes positively to aggregate productivity growth (last column). Exiting SE-P firms, however, tend to be roughly as productive as incumbent businesses. Put together, serial entrepreneurs are responsible for about 20% of overall aggregate productivity growth.

**Fig. 1:**
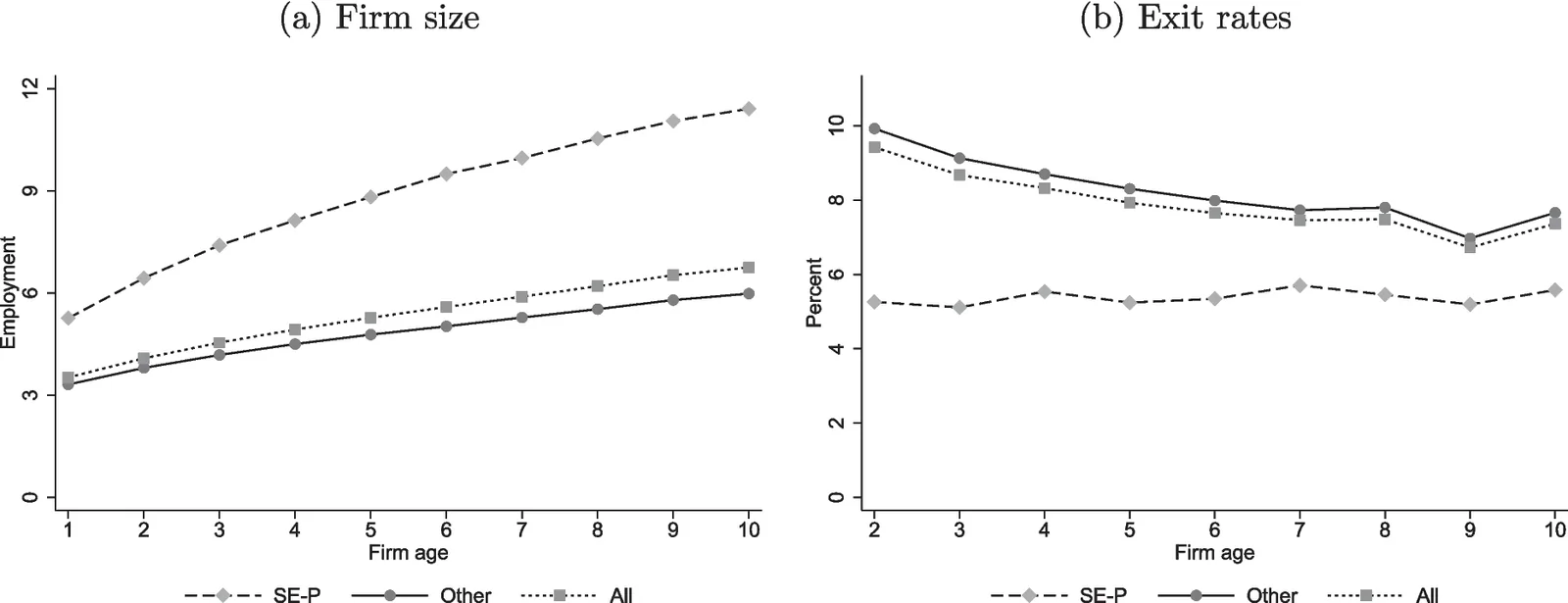
Size and exit profiles by age. Notes: the left panel shows average firm size by age, while the right panel shows average exit rates by age. Both panels depict all, other and portfolio serial entrepreneur (SE-P) businesses

#### Contributions to aggregate employment, job creation, and destruction

Next, Table 6 shows that while SE-P businesses represent less than 14% of all firms, they alone employ more than 23% of the workforce. This is consistent with our estimated premia which show that SE-P firms are considerably larger compared to other businesses.

The table further reports that SE-P firms create (and to a lesser extent also destroy) a disproportionate amount of jobs. In particular, firms of portfolio serial entrepreneurs are responsible for 23% of all job creation and about 18% of all job destruction. These values are already suggestive of a *net* job creation prowess of SE-P firms to which we turn next.

### Business dynamism

Next, we focus on how SE-P firms help shape average business dynamism patterns. In this subsection, we document life-cycle patterns of job creation, destruction, and the distribution of firm-level (net) growth rates. These variables not only underlie the aggregate job creation patterns discussed above, but they have also been shown to be important indicators of *aggregate* productivity-enhancing factor reallocation (see Haltiwanger et al., 2013).

#### Up-or-out dynamics

Figure 1 depicts life-cycle profiles for average firm size and exit rates by firm age.^16^ It does so separately for all, other and SE-P firms. As documented in Sect. 3.3, *on average* young businesses exit more often, but surviving young firms almost double in size in the first ten years of their existence. These average patterns, however, hide a dramatic difference between the life-cycle patterns of other and SE-P firms.

**Fig. 2:**
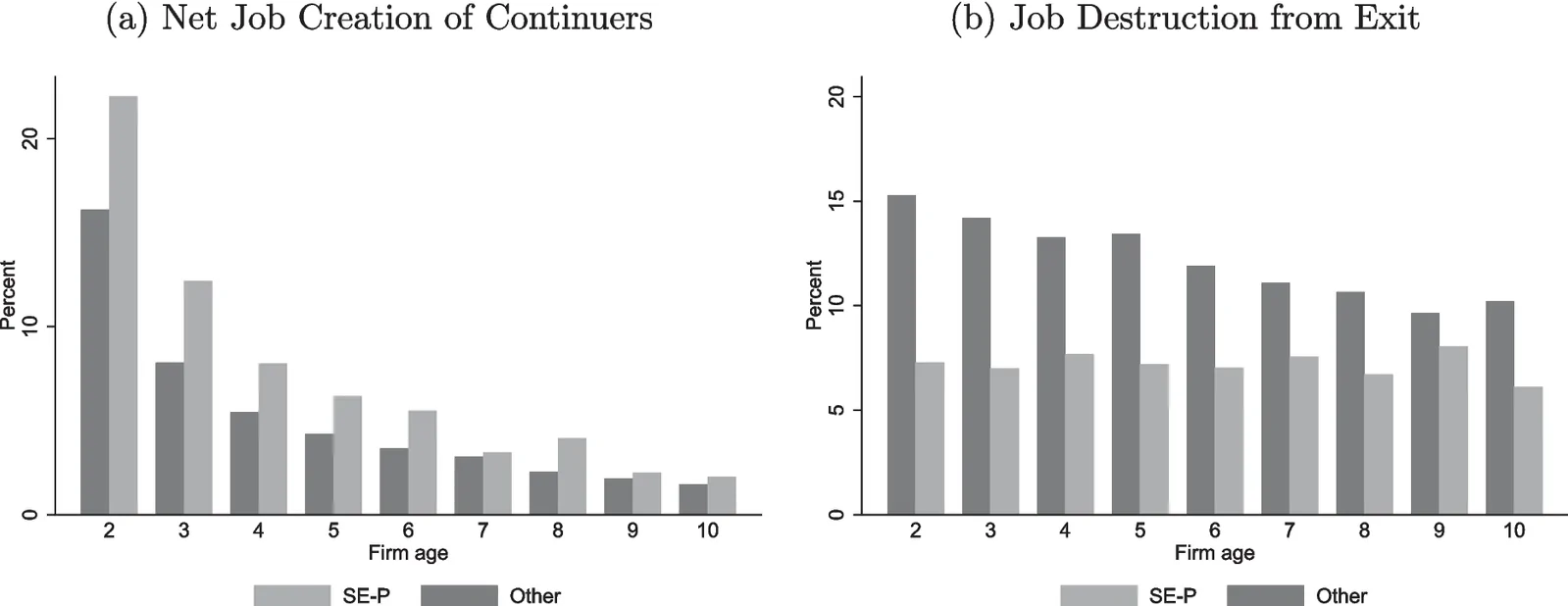
Up-or-out dynamics. Notes: the figure shows net job creation (NJC) rates of continuing businesses (**a**), and job destruction (JD) rates from exit (**b**). Both as a function of business age and separately for other and portfolio serial entrepreneur (SE-P) firms

The left panel of Fig. 1 shows that SE-P firms not only start up more than 1.5 times larger than other businesses, but they also grow by 122% (on average) within ten years of their existence. In contrast, other businesses on average grow by “only” 86% between startup and the age of 10.

The right panel of Fig. 1 shows that the rate at which SE-P firms shut down is not only considerably lower on average, but it is also essentially flat over the course of their life-cycle. Readers will recall, however, that exit patterns of SE-P firms are (at least partly) mechanically driven by the fact that portfolio serial entrepreneurship requires a certain degree of survival. We return to this point in our Discussion Sect. 6.

Finally, note that in the absence of SE-P firms, average business dynamism would resemble that of other businesses in Fig. 1. Therefore, if it were not for SE-P firms — and ignoring general equilibrium effects — 10-year-old firms would on average be more than 12% smaller and average exit rates would be about 5% (0.4 percentage points) higher.

#### Job creation, destruction, and excess reallocation

Next, Fig. 2 shows how the stark differences in up-or-out dynamics between O and SE-P firms are reflected in their job creation and destruction patterns. Specifically, the left panel of Fig. 2 depicts net job creation of continuing firms, while the right panel shows job destruction from exit. In both panels, we separately depict the values for portfolio serial entrepreneurs and other businesses.

Two patterns stand out. First, net job creation by continuing other businesses is about a fifth lower compared to that by portfolio serial entrepreneurs. This holds true essentially across the entire firm life cycle. Second, reflecting the flat exit rates of SE-P firms documented above, job destruction from exit is also essentially constant among SE-P firms. Combined, this evidence documents that SE-P firms are important drivers of aggregate business dynamism.

Interestingly, gross job creation rates are comparable between other (29%) and SE-P firms (28%). However, SE-P firms destroy fewer jobs (13%) compared to other businesses (17%). Therefore, the net job creation prowess of SE-P firms can be attributed primarily to their lower destruction rates. This, in turn, also leads to larger excess job reallocation — total job reallocation minus realized net employment growth (see Davis and Haltiwanger, 1992 — among other firms (34%) compared to portfolio serial entrepreneur businesses (26%).

**Fig. 3:**
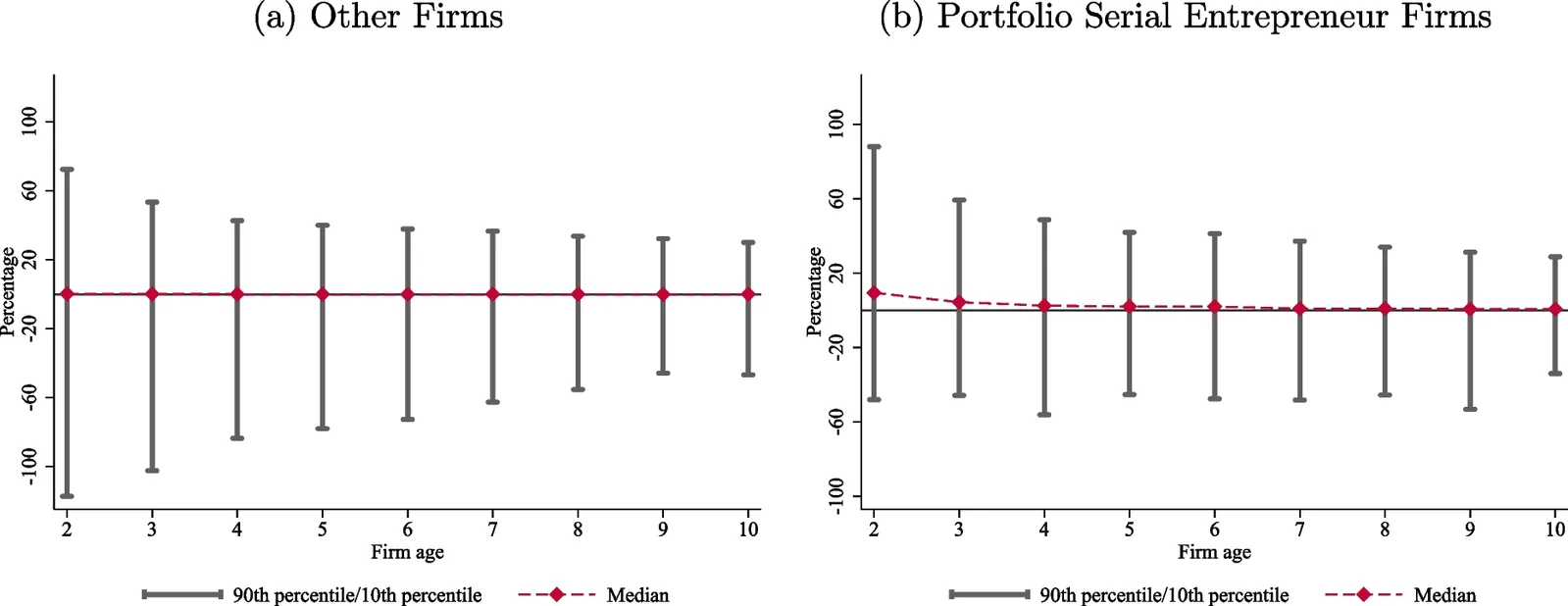
Employment growth distributions. Notes: the figure shows employment growth distributions of continuing businesses for other (left panel) and portfolio serial entrepreneur firms (right panel). Both as a function of business age and employment-weighted. Specifically, the figure depicts the 10th and 90th growth percentiles in each age category together with the median

#### Growth distributions over the life-cycle

We now zoom in on the “up-” part of business dynamism and analyze net job creation and its distribution over firms’ life-cycles. Figure 3 shows the distribution of employment growth rates among other (left panel) and SE-P firms (right panel) by age.

As is clearly visible from the figure, the entire growth distribution of SE-P firms is shifted towards faster growth rates, compared to other firms — and this holds true throughout firms’ life-cycles. Specifically, the lower end (10th percentile) of the growth distribution among other firms ranges between about -120% and -50%.^17^ In contrast, the 10th percentile among SE-P firms hovers around -50%. In addition, the upper end (90th percentile) of the growth distribution is considerably higher for SE-P firms, especially early on in firms’ life-cycles.

These two effects combined give rise to a higher median growth rate of SE-P firms.^18^ Specifically, the median SE-P firm grows at a rate of about 15% at age 2. Even at age 10, the median SE-P firm displays a slightly positive growth rate. In contrast, the median other business essentially does not grow — independent of its age.

### Serial entrepreneur firms and gazelles

The previous paragraphs documented the growth pro-wess of SE-P firms. We now turn our attention to quantifying the extent to which this is driven by a prominent sub-group of businesses — high-growth firms, so-called “gazelles.” This focus builds on established evidence that while rare, gazelles are important for aggregate outcomes (see Henrekson and Johansson, 2010; Haltiwanger et al., 2016). In the Appendix, we report what these patterns imply for the broader (dynamics of the) firm size distribution.

**Table 7:** Contribution of high-growth firms to aggregates (in %)

	All		Other	SE-P
Firms	8.6		70.9	29.1
Employment	31.1		67.3	32.7
Job creation	44.2		67.8	32.2

Notes: the table reports characteristics of all high-growth firms (first column) and those owned by “other” entrepreneurs and portfolio serial entrepreneurs (“SE-P”). Shares are in % of all businesses in the first column, while they are a fraction of all high-growth firms in the second and third columns (hence, shares for other and SE-P gazelles add to 100%)

#### Serial entrepreneur firms and gazelles

To begin with, Table 7 confirms the findings in Henrekson and Johansson (2010) and Haltiwanger et al. (2016) that gazelles contribute disproportionately to aggregate employment and job creation also in Portugal. In particular, the first column of Table 7 shows that while only about 9% of all firms in Portugal can be classified as gazelles according to the Eurostat-OECD definition, these firms alone account for almost a third of employment and more than 40% of newly created jobs in the entire economy.

The second and third columns of Table 7 then show the contributions of other and portfolio serial entrepreneur gazelles to the overall patterns of high-growth firms. Most importantly, the table documents that about 29% of all high-growth firms are owned by portfolio serial entrepreneurs. Given that SE-P firms account for less than 14% of all firms, this implies that portfolio serial entrepreneurs are almost *three times more likely* to own a gazelle compared to other business owners.^19^

**Table 8:** Serial entrepreneur premium: high-growth firms

	Other	SE-P	SE-P Premium
Size (workers)	23.3	28.4	$$0.15^{***}$$
Exit (in %)	5.4	3.9	$$-1.37^{***}$$
Growth (in %)	14.4	13.1	0.35
Productivity (agg.=1)	0.86	1.13	0.06

Notes: the columns show, respectively, the averages of other and SE-P high-growth firms and the SE-P premium estimated from regression Eq. 5. The rows depict, respectively, average (employment) size, exit rates, (employment-weighted) net employment growth, and average labor productivity scaled by labor productivity of all firms. Ordinary Least Squares estimates with robust standard errors clustered at the firm level in parentheses. *** stands for statistical significance at the 1% level

#### Gazelles owned by other and portfolio serial entrepre-neurs

While portfolio serial entrepreneurs are about three times more likely to own a gazelle, the performance of high-growth firms owned by portfolio serial entrepreneurs is only slightly superior to that of other gazelles.

In particular, Table 8 shows estimated serial entrepre-neur premia for the sub-group of high-growth firms. The table documents that gazelles of portfolio serial entrepreneurs are 15% larger and about 25% less likely to shut down. In contrast, there are no statistical differences between gazelles owned by other and portfolio serial entrepreneurs concerning growth rates and productivity.^20^

## Potential driving forces

As a final part of our analysis, we offer an investigation of a range of potential sources underlying the superior performance of SE-P businesses and, in turn, their disproportionate aggregate contribution. In particular, we focus on — loosely speaking — “learning” (see, for example, Cope, 2005; Politis, 2005; Lazear, 2005; Eggers and Song, 2015; Lafontaine and Shaw, 2016 and “selection” (see, for example, Ucbasaran et al., 2008; Sedláček and Sterk, 2017; Shaw and Sørensen, 2022; Sanguineti et al., 2025) as two broad channels driving firm performance.

We interpret both terms broadly. In particular, while “learning” may explicitly include improvements in the productivity of business owners, it may also cover other ex-post or dynamic forces. An example of the latter includes for instance the gradual accumulation of net worth. In contrast, “selection” focuses on the various ex-ante factors on which serial entrepreneurs (and their firms) may be selecting. These may include entrepreneur characteristics or ability, characteristics of the workforce employed in their firms or financial factors.

While a causal analysis is beyond the scope of this paper, the results below provide a guide for theories attempting to model and understand serial entrepreneurship as well as shed light on the sources of firm success more generally. A fruitful avenue for future research may be the development of a structural model of serial entrepreneurship or an empirical framework with novel exogenous variation enabling to more decisively pin-point causal relationships.^21^

**Fig. 4:**
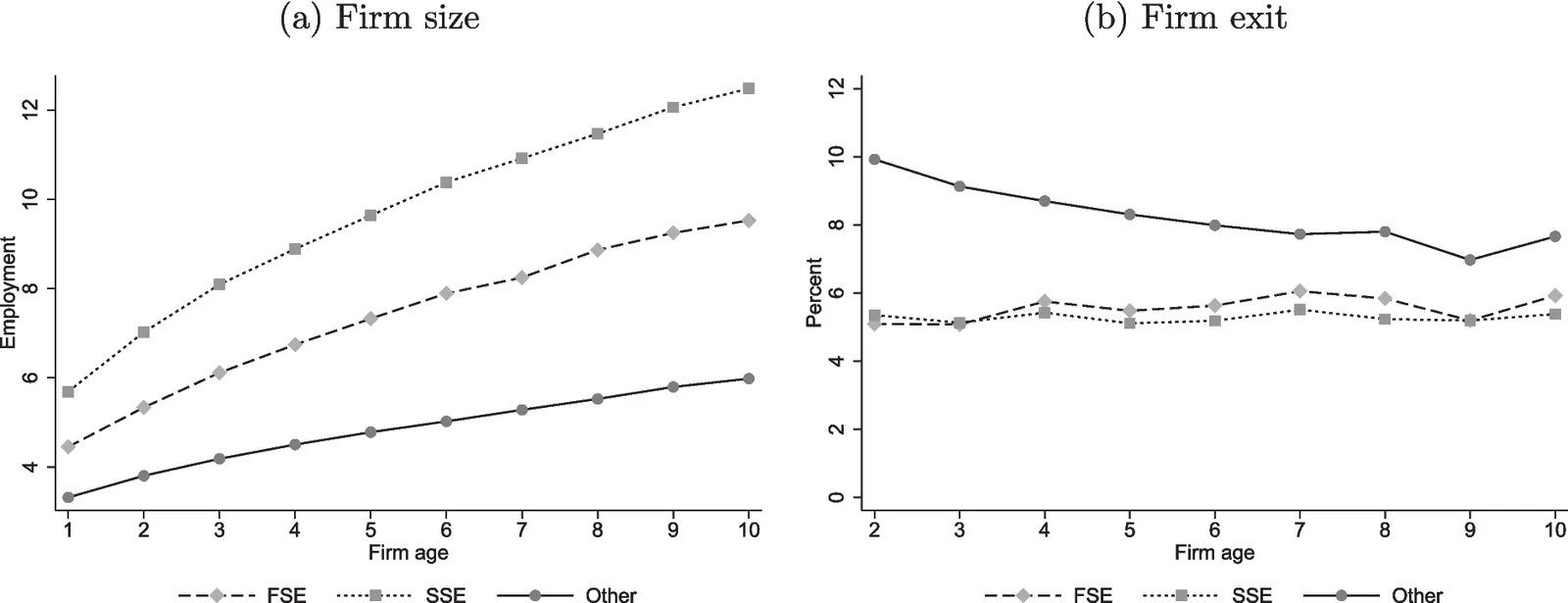
Size and exit profiles by age: other, first SE-P, and subsequent SE-P firms. Notes: the left panel shows average firm size by firm age, while the right panel shows average exit rates by firm age. Both subpanels depict other and portfolio serial entrepreneur businesses, where the latter are split into first (FSE) and subsequent (SSE) businesses of portfolio serial entrepreneurs

### Learning and ex-post factors

As a first potential driver of the serial entrepreneur premia, we consider learning and other ex-post factors. Our data offers a natural way to disentangle ex-ante factors from ex-post developments by separately analyzing the performance of “first” (FSE) and “subsequent” (SSE) businesses of portfolio serial entrepreneurs.

Intuitively, FSE firms are those businesses which entrepreneurs owned *before* they could be classified as portfolio serial entrepreneurs. In contrast, SSE businesses are the cause of the serial entrepreneur classification and constitute their second and further firms. Under the presumption of learning by business owners or the (favorable) influence of other ex-post factors, subsequent firms of portfolio serial owners should be characterized by superior performance over and above their “first” business.^22^

**Table 9:** FSE and SSE premia

				Premia		
	Other	FSE	SSE	FSE-O	SSE-O	SSE-FSE
Size (workers)	5.4	9.3	11.9	$$0.37^{***}$$	$$0.54^{***}$$	$$0.24^{***}$$
Exit (in %)	8.0	5.6	5.4	$$-1.56^{***}$$	$$-2.11^{***}$$	$$0.16^{***}$$
Growth (in %)	8.9	10.0	9.7	$$2.02^{***}$$	$$1.68^{***}$$	$$-0.46^{{***}}$$
Productivity (agg.=1)	0.82	1.13	1.19	$$0.19^{***}$$	$$0.24^{***}$$	0.04

Notes: the first three columns show, respectively, the averages of other, first, and subsequent SE-P firms. Columns 4 to 6 show, respectively, premia estimated from Eq. 8: “FSE-O” is the premium of first portfolio serial entrepreneur businesses over other firms, “SSE-O” is the premium of subsequent portfolio serial entrepreneur businesses over other firms, and “SSE-FSE” is the premium of subsequent over first SE-P firms. The rows depict, respectively, average size (employment), exit rates, (employment-weighted) size growth, and firm-level labor productivity scaled by labor productivity of all businesses. Ordinary Least Squares estimates with robust standard errors clustered at the firm level in parentheses. *** stands for statistical significance at the 1% level

#### Life-cycle patterns and estimated premia

Figure 4 depicts the life-cycle profiles of firm sizes (left panel) and exit rates (right panel) for other and SE-P firms. As opposed to Fig. 1, average size and exit rates of SE-P firms are split into those pertaining to the first and subsequent businesses. The figure paints a clear picture — both first and subsequent firms of portfolio serial entrepreneurs display noticeably superior life-cycle patterns compared to those of other businesses. In addition, subsequent businesses of portfolio serial entrepreneurs substantially outperform their first businesses when it comes to firm size (left panel of Fig. 4).

To formally test these patterns, we re-estimate our serial entrepreneur premia for these groups of firms. Specifically, we consider the following regression

8 $$\begin{aligned} y_{i,s,t} = \alpha + \beta 1\!\!1_{\text {s,s\_comp}} + \delta F_{i,s,t} + \epsilon _{i,s,t}, \end{aligned}$$

where $y_{i,s,t}$ is again a given outcome variable of interest (log employment, exit rates, net employment growth and log labor productivity) for firm *i* in year *t* and in a given group of firms $s \in \{\text {O},\text {FSE},\text {SSE}\}$. In a given regression, however, we always restrict the sample to only two mutually exclusive groups — a base group *s* and a comparison group $s_{\text {comp}}$. The variable $1\!\!1_{\text {s,s\_comp}}$ is an indicator function, which depends on the given base and comparison groups. This indicator function is equal to one when firm *i* belongs to group *s*, and it is zero otherwise. Finally, in regression Eq. 8, we again control for age, industry, and year fixed effects ($F_{i,s,t}$).

Table 9 shows the results where columns 1 to 3 depict average values of size, exit, growth rates, and labor productivity in the three groups of firms. Columns 4 to 6 then report the coefficients β in the various versions of regression Eq. 8. The results document that both FSE and SSE firms are larger, exit less frequently, grow faster, and are more productive relative to other businesses (columns 4 and 5).

However, the difference in performance between FSE and SSE firms is less clear. While SSE firms are larger, conditional on other observables, they tend to exit more frequently and growth somewhat slower. Therefore, while learning and other ex-post factors may contribute to the success of serial entrepreneur firms, other forces are also likely at play.^23^

### Selection and ex-ante factors

We now turn to analyzing selection as an additional source of S-P premia. As with learning, we interpret selection more generally as reflecting a range of ex-ante factors which have been shown to be important for firm performance, see, e.g., Sterk et al. (2021).

#### Estimation

In this analysis, we consider three groups of variables that plausibly explain the serial entrepreneur premia we find: (1) entrepreneur characteristics, (2) firm workforce characteristics, and (3) firm financial condition. To estimate the impact of these factors on firm performance, we once again revisit our serial entrepreneur premia regressions (between other (O) and portfolio serial entrepreneur (SE-P) firms):

9 $$\begin{aligned} y_{i,t} = \alpha + \beta 1\!\!1_{i\in SE} + \gamma F_{i,t} + \delta G_{i,t} + \epsilon _{i,t}, \end{aligned}$$

where $G_{i,t}$ refers to our explanatory variables. Table 10 shows the results. Given our focus on business owners, the first four columns report estimates for the case when we consider entrepreneur characteristics as the only set of explanatory factors. The remaining columns show results for the case where we, in addition, consider characteristics of the workforce in SE-P firms and their financial condition.

The first row of Table 10 estimates “unconditional” serial entrepreneur premia, $\beta ^u$, i.e., those estimated in Eq. 5 which ignore the explanatory variables.^24^ The second row reports “conditional” serial entrepreneur premia, $\beta ^c$, estimated in Eq. 9 which take the explanatory factors, $G_{i,t}$, into account. The remaining rows report the contributions of individual explanatory factors to the estimated SE-P premia following the Gelbach (2016) decomposition, which is invariant to the “order of elimination” of regressors. Within each panel, the first line reports the “total contribution” of the given set of factors. The second line shows the total contribution as a share of the unconditional SE-P premium, $\beta ^u$. Finally, the remaining rows of each panel report contributions of the underlying individual factors.

**Table 10:** Drivers of portfolio serial entrepreneur premia

	Entrepreneur characteristics only					Entrepreneur & other characteristics			
	Size	Exit	Growth	Productivity		Size	Exit	Growth	Productivity
Unconditional SE-P premium, $\beta ^u$	0.46	-1.51	1.64	0.30		0.46	-0.40	0.52	0.28
Conditional SE-P premium, $\beta ^c$	0.34	-0.80	1.03	0.16		0.33	0.04	0.00	0.15
A: Entrepreneur characteristics									
Total contribution	0.12	-0.71	0.61	0.14		0.10	-0.18	0.23	0.10
Share of SE-P premium	27%	47%	37%	46%		22%	43%	45%	35%
Contributing factors:									
*- age*	-0.01	-0.06	-0.09	0.00		-0.00	-0.02	-0.06	-0.00
*- gender*	0.00	-0.04	0.04	0.01		-0.00	-0.03	0.05	0.01
*- education*	0.02	-0.26	0.29	0.05		0.02	-0.17	0.12	0.03
*- owner/manager*	0.01	-0.21	0.03	0.00		-0.00	-0.02	0.03	0.00
*- past wages*	0.06	0.07	0.23	0.05		0.06	0.15	0.09	0.05
*- management experience*	0.04	-0.22	0.11	0.02		0.02	-0.08	0.01	0.01
B: Workforce characteristics									
Total contribution						0.00	-0.05	0.17	0.01
Share of SE-P premium						0%	13%	33%	3%
Contributing factors:									
*- age*						0.01	-0.06	0.14	0.00
*- gender*						0.00	-0.00	-0.00	0.00
*- education*						-0.01	0.00	0.03	0.01
C: Financial factors									
Total contribution						0.02	-0.21	0.11	0.02
Share of SE-P premium						5%	52%	22%	7%
Contributing factors:									
*- liquidity*						0.00	-0.01	0.01	0.00
*- solvency*						0.01	-0.23	0.10	0.01
*- cash ratio*						0.01	0.03	-0.00	0.00

Notes: the table reports results from estimating Eq. 9. The first row reports “unconditional” SE-P premia (between SE-P and O firms), $\beta ^u$, which ignore explanatory factors, $G_{i,t}$. The second row shows conditional SE-P premia, $\beta ^c$, which control for $G_{i,t}$. The remainder of the table reports individual contributions of explanatory factors, grouped into “Entrepreneur characteristics” (Panel A), “Workforce quality” (Panel B), and “Financial factors” (Panel C). Each panel reports the “Total contribution” of the set of factors, “Share of SE-P premia” defined as (Total contribution)/$\beta ^u$ and the contributions of the underlying individual factors. The table reports results for a decomposition using only entrepreneur characteristics (first 4 columns) and all considered factors (last 4 columns)

#### Results: entrepreneur characteristics

The results in the first four columns of Panel A in Table 10 suggest that entrepreneur characteristics alone can explain between 20 and almost 50% of the estimated (unconditional) serial entrepreneur premia. Similar contributions can be found also when conditioning on other explanatory factors (last four rows in Panel A).

The three most important contributors in this regard are education, past wages and past managerial experience. While past wages can be viewed as a proxy of entrepreneurial ability, managerial experience (in past employment) has been identified as an important factor determining entrepreneurs’ success (see, e.g., Westhead et al., 2005).^25^ Recall that portfolio serial entrepreneurs outperform novice (and sequential serial entrepreneurs) in all these dimensions, see Table 3.

These findings are consistent with existing research, which shows that selection on entrepreneurs’ ability and education is an important determinant of entrepreneurship and subsequent firm success (see, e.g., Ucbasaran et al., 2003; Chen, 2013; Carbonara et al., 2020; Queiró, 2022).

Other entrepreneur characteristics do not provide quantitatively strong contributions to the estimated serial entrepreneur premia. Age contributes positively to the exit premium but negatively to the growth premium. This may be interpreted as older portfolio serial entrepreneurs tending to run more stable businesses with less upside potential. Finally, while being an owner-manager can help explain lower exit rates of SE-P firms, it does not seem to be quantitatively important for the other estimated SE-P premia.^26^

#### Results: workforce characteristics

As a second set of explanatory factors, we consider characteristics of employees working in SE-P firms. The results suggest that the composition of the workforce is related to the growth and exit patterns of SE-P firms. In particular, Panel B of Table 10 shows that workforce characteristics account for about 13% of the exit premium and 33% of the growth premium.

In particular, employee age — and to a lesser extent education — play an important role in this regard. As such, these results connect to relatively sparse existing research studying how the composition of labor supply (especially in aging populations) may affect business dynamism (see, e.g., Engbom, 2019; Ignaszak, 2020). Finally, workforce characteristics do not seem to have any bite when it comes to firm size and productivity premia.

#### Results: financial factors

Finally, Panel C of Table 10 reports the contributions of financial factors to the estimated serial entrepreneur premia. While financial factors contribute only modestly to size and productivity premia of serial entrepreneurs, they play an important role in understanding the advantage of serial entrepreneurs when it comes to firm exit and growth. In particular, financial factors account for between 20 and 50% of the estimated growth and exit premia, respectively.

These effects are dominantly driven by differences in solvency between portfolio serial and other entrepreneur firms. In particular, businesses of portfolio serial entrepreneurs enjoy a liability-to-assets ratio which is about 1/4 lower than that of other businesses. In other words, considerably lower indebtedness of serial entrepreneur firms goes a long way in explaining their superior performance. This points to the importance of financial constraints as key determinants of firm exit and growth, consistent with existing research (see, e.g., Parker, 2013; Carbonara et al., 2020).

In the Appendix, we repeat our estimation for the sub-group of first businesses of portfolio serial entrepreneurs. The results suggest that while entrepreneur and workforce characteristics display largely similar contributions to the estimated premia of FSE firms, the positive effect of financial factors is dampened. This suggests that portfolio serial entrepreneurs seem to enter entrepreneurship with a stronger financial position compared to other entrepreneurs. At the same time, and consistent with our results on “learning,” this financial edge gets only stronger for their subsequent firms. Therefore, SSE firms seem to benefit from the initial success of their FSE predecessors.

## Discussion

In this section, we provide a discussion of our results. In doing so, we not only put our findings in perspective with existing research, but also point out some shortcomings of our data and analysis, which pave the way for future research.

### Right censoring and M&As

We begin by discussing several properties of our data which may affect the results. First, our results may be biased because of right censoring — a typical concern when studying serial entrepreneurship. Specifically, business owners may be classified as “novice” entrepreneurs, despite the fact that they will start a second business in the future, *outside* our sample period. The Appendix provides robustness checks using a truncated sample and argues that this feature only likely creates a downward bias in our estimates of the importance of portfolio serial entrepreneur businesses.

Second, mergers and acquisitions (M&A) can bias the macroeconomic contribution of SE-P firms. Intuitively, expansions (or entry) of SE-P firms via M&A activity should not be counted as contributions to aggregate job creation since they are exactly offset by the exit of the acquired business.^27^ However, Mata and Portugal (2004) report that M&As account for less than 1% of all business shutdowns. In addition, we investigate M&As further by proxying them with startups in which some workers report having tenure larger than 1 year. This proxy shows that, for instance, at the end of our sample in 2017, only about 3% of new firms would be classified as M&As.^28^

### Selection and learning

Studying serial entrepreneurship implies focusing on an endogenously selected group of businesses. Intuitively, SE-P firms are more likely to display lower exit rates because the first businesses of serial entrepreneurs need to survive before the owner starts another firm. While such selection may mechanically deliver “serial entrepreneur premia,” it remains an open question whether SE-P businesses contribute quantitatively to macroeconomic outcomes — precisely the focus of our paper. In this sense, our approach is analogous to the study of the macroeconomic impact of “high-growth” firms, which relies on identifying such businesses based on observed high-growth outcomes (see, e.g., Haltiwanger et al., 2016).

Aside from the above, selection may also blur the interpretation of our results pertaining to the effects of learning on the performance of subsequent businesses of portfolio serial entrepreneurs. In particular, the performance of subsequent firms may be affected by selection on outcomes of the first businesses owned by portfolio serial entrepreneurs. Intuitively, initial success of the first SE-P firm may provide an improved financial position which can help kickstart subsequent SE-P firms (see, e.g., Brandt et al., 2025, for a model of serial entrepreneurship with financial frictions).

### Drivers of serial entrepreneur premia

As we discussed already in Sect. 2, we are not the first to study the determinants of serial entrepreneurship (see, e.g., Wright et al., 1998; Ucbasaran et al., 2008; Carbonara et al., 2020). Our findings from Sect. 5 closely relate to some of the existing work. For example, the importance of financial conditions and skill for (re-)entering entrepreneurship has also been documented in Parker (2013); Carbonara et al. (2020). Education has also been highlighted as a key determinant of entrepreneur success in, e.g., Ucbasaran et al. (2008) or Queiró (2022). Aside from our macroeconomic focus, this paper contributes to existing findings through a key advantage of our data — near universal coverage of workers, firms, and their owners in the Portuguese economy.

That said, existing research also highlights a range of other — psychological and societal — factors which are important for (success in) entrepreneurship (see, e.g., Westhead and Wright, 1998; Ucbasaran et al., 2003; Carbonara et al., 2020). Our data cannot speak to such drivers, pointing to an important avenue for future research.

### Entrepreneurship and macroeconomic models

A series of influential papers have documented that young firms, and in particular a rare group of high-growth gazelles, contribute disproportionately to aggregate job creation and productivity growth (see, e.g., Haltiwanger et al., 2016; Decker et al., 2020). While such firm heterogeneity has often been largely attributed to transitory post-entry productivity or demand differences (see, e.g., Hopenhayn and Rogerson, 1993, for a seminal contribution), there is growing evidence that also differences present at the entry phase (ex-ante heterogeneity) can have long-lasting effects on firms and, in turn, shape aggregate dynamics (see, e.g., Jovanovic, 1982; Melitz, 2003; Sterk et al., 2021). Our results, therefore, constitute a further step towards a better understanding of the micro-level sources and macroeconomic impact of firm-level heterogeneity.

In particular, while Sterk et al. (2021) recently documented that ex-ante firm heterogeneity is crucial for understanding aggregate dynamics, our results suggest that serial entrepreneurship may be one such ex-ante characteristic. Studying these patterns further (both empirically and theoretically) points towards a fruitful avenue for future research. In particular, considering occupational choice settings in combination with worker and entrepreneur heterogeneity, which then (co-)determines differences in firm performance, may be a particularly interesting framework to study in the future.

## Concluding remarks

Using unique administrative data, this paper documents novel facts about the *macroeconomic* importance of serial entrepreneurs — business owners who simultaneously own multiple firms. In particular, serial entrepreneur firms (i) contribute disproportionately to aggregate job creation and productivity growth, (ii) shape overall business dynamism, and (iii) are more likely to exhibit high-growth over their life-cycle. In addition, we provide a novel analysis of the potential sources of such superior performance. Our results point especially towards selection on entrepreneur education, ability, and financial conditions as the key determinants of success.

This paper contributes to the existing literature in two distinct ways. First, it makes a strong case for the analysis of serial entrepreneurs from a *macroeconomic* perspective — a topic that is not well understood. This is true not only for empirical work, which has been held back by a lack of available data sources, but especially for theoretical and quantitative macroeconomic research from which serial entrepreneurs are effectively completely missing. Second, it sheds light on the potential reasons behind firm success, guiding the development of new theories and providing a means to discipline existing macroeconomic models of business dynamism.

Our results also open the door to further empirical questions, which are, however, beyond the scope of this paper. Most importantly, obtaining exogenous variation in the data or developing a structural model of serial entrepreneurship to uncover causal relationships between individual or firm characteristics and subsequent business performance would be particularly interesting.

## Supplementary Information

Below is the link to the electronic supplementary material.Supplementary file 1 (pdf 1506 KB)

## Data Availability

This article uses *confidential administrative data* from the Portuguese Ministry of Employment, Solidarity and Social Security. This prevents us from fully sharing all our data.

## Footnotes

1 The next sections discuss in detail how our classification relates to the existing literature (see, e.g., Wright et al. 1998; Carbonara et al. 2020) and we provide results also for other classes of entrepreneurs.

2 We define gazelles according to the Eurostat-OECD definition (see European Commission 2007) as young businesses which report average annual growth rates above 20% for at least three consecutive years. The Appendix shows that similar results are obtained with alternative definitions of gazelles which follow Decker et al. (2020).

3 This result is consistent with evidence from Denmark focusing on young serial entrepreneurs (see Shaw and Sørensen 2022).

4 These findings are consistent with other work suggesting education and selection on ability and past managerial experience as important for firm dynamics (see, for example, Robinson and Sexton 1994; Davidsson and Honig, 2003; Westhead et al., 2005; Chen, 2013; Queiró, 2022; Krieger et al., 2022.

5 This terminology ignores the fact that “novice” entrepreneurs may be running their business for many years and, therefore, be relatively experienced business owners.

6 Without focusing on the macroeconomic context, Parker (2014) provides an occupational choice model for (serial) entrepreneurship.

7 This feature of the QP is rare. For instance, Choi et al. (2021) use U.S. Census Bureau data and studies the role of “founding teams” for the performance of young firms. In their data, however, founders of the majority of corporations are not directly observable (though they can be proxied).

8 This classification is defined by Portuguese law (Lima and Pereira, 2003) . See also Queiró (2022) for a related study of managers in Portugal.

9 The Appendix shows that our results are robust to restricting the analysis to single-owner businesses and to adopting a time-varying definition of serial entrepreneurship, which re-categorizes business owners each year based on the number of firms they own in a given year. Note further that 93% of SE-P firms are owned fully by portfolio serial entrepreneurs (i.e., only 7% of SE-P businesses also have a non-portfolio entrepreneur co-owner). Finally, some studies (see, e.g., Westhead and Wright 1998) further restrict portfolio entrepreneurs to own their original, very first, business. In our sample, the majority (about 70-80%) of portfolio serial entrepreneurs still own their very first business.

10 Similar results are obtained when focusing on businesses which are entirely owned by serial entrepreneurs.

11 In the Appendix, we show that our results are similar when using alternative definitions of high-growth firms (see, e.g., Haltiwanger et al. 2016).

12 The exception seems to be “Real estate and other activities” in which portfolio serial entrepreneurship is more prevalent.

13 We note that the SE premium, β, is not restricted to be positive and can.

14 In regard to the firm exit, the serial entrepreneur premium is estimated at about 1.7 percentage points. This is about 21% of the unconditional average exit rate of 8% among novice businesses. Similarly, in the case of firm growth, the serial entrepreneur premium is estimated at 1.7 percentage points, which is about 20% of the unconditional average growth rate of 8.9% among novice firms.

15 We note that not all studies of serial entrepreneurs find that their firms enjoy superior performance. For instance, using a sample of 354 Scottish firms, Westhead et al. (2005) do not find any difference in performance between novice and serial entrepreneur firms.

16 Fig. 1 reports “raw” numbers. In the Appendix, we show that these results are similar when controlling for the industry composition and geographical location of O and SE-P firms.

17 Recall the definition of employment growth follows the Davis et al. (1996) methodology in which firm exit is associated with -200% employment growth.

18 In the Appendix, we further show that higher growth of SE-P firms is due to both a faster intensive margin (within-establishment growth) and extensive margin (growth in the number of establishments per firm).

19 The probability that a firm of a particular group of entrepreneurs, $i\in \{O,SE-P\}$, is a gazelle can be expressed as $Pr(\text {gazelle}|i)=\frac{\# \text {gazelles}}{\#\text {all firms}} \times \frac{\# \text {i-type gazelles}}{\# \text {gazelles}}/(\frac{\# \text {i-type firms}}{\# \text {all firms}})$. For other and portfolio serial entrepreneurs, these values are, respectively, $Pr(\text {gazelle}|R)=0.09\times 0.71 /0.86 \approx 0.07$ and $Pr(\text {gazelle}|SE)=0.09\times 0.29 /0.14 \approx 0.19$.

20 The Appendix shows that a similar picture is provided by the life-cycle patterns of portfolio serial entrepreneurs and other gazelles.

21 See Brandt et al. (2025) for a theory rationalizing how the behavior of serial entrepreneurs is driven by ability and financial frictions, as well as empirical evidence on serial entrepreneurs from China.

22 In the data, it takes — on average — owners almost 6 years to get involved in their subsequent business (and therefore qualify to be categorized as a serial owners). There is, however, a large degree of heterogeneity in this regard. While the “fastest” 10% of serial entrepreneurs start their subsequent businesses within a year, the “slowest” 10% do so after about 12 years.

23 Recent evidence from Denmark also suggests that sales of second businesses of portfolio serial entrepreneurs are larger than those of their first firms, see Shaw and Sørensen (2022). Similarly, using Texas retail firms, Lafontaine and Shaw (2016) show that — after controlling for individual fixed effects — experience of serial entrepreneurs matters for the success of their businesses.

24 Note that since merging the QP with SCIE results in a somewhat different sample of firms, the estimated SE-P premia are not identical in the first and last four columns of Table 10. For the same reason, the unconditional premia are also not identical to those reported in Table 4. Further note that as a result of rounding in each cell, the sum of individual factors does not always equal to the total contribution.

25 Note that past wages contribute negatively to the estimated exit premium. We interpret this as an indication of better outside options of entrepreneurs who previously earned high wages.

26 Note that this does not imply that managerial input is not important for firm dynamics. Rather, it suggests that serial entrepreneurs are not too different from other businesses in this regard.

27 In the data, the unique identifiers of the firms involved in the M&A operation are transmitted to the acquiring firm, while the identifiers of the acquired firms disappear. Therefore, the acquired businesses are counted as exits in the data.

28 This fraction is about 6% among SE-P firms. Estimating SE premia separately for these firms shows that both “M&A” and “non-M&A” SE-P firms outperform other businesses.
