# Supplementary material for: Serial entrepreneurs and the macroeconomy
Source: Small Bus Econ (Dordr). 2026 Apr 22;67(2):995–1017. doi: 10.1007/s11187-026-01213-8 (PMC13521969; doi:10.1007/s11187-026-01213-8)
Supplement: Supplementary file 1 [file 11187_2026_1213_MOESM1_ESM.pdf]

Online Appendix to "Serial Entrepreneurs and the Macroeconomy"

Table of Contents

A Data **1**

A.1 Identifying Serial Entrepreneurs and Their Firms . . . 3

A.2 Outcome variables . . . 6

B Additional Empirical Results **8**

B.1 Alternative measurement of serial entrepreneurs . . . 8

B.2 Sequential vs Portfolio Entrepreneurs . . . 11

B.3 Analysis on a truncated sample period . . . 13

B.4 Sole ownership only . . . 16

B.5 Alternative definition of high-growth firms . . . 18

B.6 Productivity Decomposition Among Continuing Firms Only . . . 19

B.7 Controlling for Industry and Location . . . 20

B.8 Margins of growth . . . 22

B.9 Implications for the Firm Size Distribution . . . 24

B.10 Life-Cycle Patterns of Gazelles . . . 26

B.11 Selection: Robustness Exercises . . . 28

A Data

Our main data source is Quadros de Pessoal (QP), a Portuguese census of private sector employees conducted each October by the Ministry of Employment, Solidarity and Social Security (MESSS). It is an extremely rich administrative employer-employee

matched panel dataset with information at the firm, establishment and individual levels. A unique advantage of the QP for our purposes is that it contains information not only on workers, but also on business *owners*.

The survey covers the universe of firms with at least one employee and their workers.[1] It is conducted on an annual basis and our sample runs from 1986 to 2017. Reporting into the QP is mandatory for all businesses that have at least one paid employee as of the survey reference week. Moreover, by law, the questionnaire needs to be available in a public space at the establishment. The administrative nature of the data and its public availability implies a high degree of coverage and reliability.

Firm Entry, Exit and Longitudinal Linkages. The QP dataset is longitudinal in nature. Each firm or establishment entering the database is assigned a unique identifying number by the MESSS. In addition, the MESSS carries out other control checks to make sure that the units which have previously reported in the database are not assigned a different identification number.

Business entry (birth) is then defined as a new identifying number which was not present in the survey in the preceding year. This definition applies to both firms and establishments, given that the two are tracked separately in the survey. Symmetrically, business exit (death) is defined as an identifying number in the preceding year which no longer exists in the current year.[2]

Thanks to these longitudinal linkages inherent to the database, we are able to connect firm-level characteristics with an individual business owner and track owners, their businesses and the workers within these businesses over time.[3] This, in turn, allows us to separately characterize firm dynamics of businesses with different owners. As will become clear below, we focus on the distinction between "serial" entrepreneurs – owners of multiple businesses – and all "other" business owners. Within the group of serial entrepreneurs, we will distinguish between those who own multiple businesses simultaneously (portfolio serial entrepreneurs) and those that do not (sequential serial entrepreneurs).

[1]The data does not contain information on civil servants, self-employed and workers who are unemployed or out of the labor force in the survey week. For our analysis, we also drop businesses from the agricultural sector, where coverage is low.

[2]This way of tracking entry, continuation and exit of businesses aligns with similar datasets in other countries, for example such as that in the Longitudinal Business Database of the U.S. Census Bureau (see for example Jarmin and Miranda, 2002). Note that businesses in the QP also report a founding date. However, cross-checking such founding dates with reported activity in the survey (and business birth as defined above) reveals many discrepancies and we, therefore, do not use this information for measuring firm entry.

[3]This feature of the QP is rare. For instance, Choi et al. (2021) use U.S. Census Bureau data and study the role of "founding teams" for the performance of young firms. In their data, however, founders of the majority of corporations are not directly observable (though they can be proxied).

Individual and Business Characteristics. Over our 1986 - 2017 sample period, we have information on roughly 2 million workers who are observed between one and thirty times, with roughly 200,000 unique firm identifiers for their jobs in the survey week.[4] The firm-level information contained in our dataset includes the sector of economic activity, geographical location, legal structure, employment, gross sales and founding year.

For some of our analysis in Section 5, pertaining to firms' financial factors, we merge our QP data with information in Sistema de Contas Integradas das Empresas (SCIE). The latter contains firm-level information on balance sheets and income statements. To preserve the coverage and definition of variables, we focus on the years 2010 - 2017 when using the SCIE.

Finally, at the worker level the QP has information on age, gender, education, occupation, date of hire, salary, job title and hours of work. Crucial to our study is a unique variable – situação profissão ("professional status") – which identifies an individual as either an owner of a business, a salaried worker, or both.

A.1 Identifying Serial Entrepreneurs and Their Firms

We now describe how we measure the key concept of this paper – serial entrepreneurship. As explained in the previous subsection, the QP dataset is uniquely equipped for this purpose. Specifically, using the "professional status" variable in the QP, we identify *business owners* as individuals who own a given firm.

"Serial" Entrepreneurs. We identify serial entrepreneurs as individuals who – at any given point in our sample – own some fraction of more than one firm. The group of serial entrepreneurs can be further categorized into (i) sequential and (ii) portfolio serial entrepreneurs.

Sequential serial entrepreneurs are individuals who experience gaps between business ownership, that is individuals who cease to be involved in a business before obtaining ownership of another. In contrast, portfolio serial entrepreneurs are individuals who own multiple businesses at the same time. As we explain below, in our baseline analysis we focus on portfolio serial entrepreneurs. Therefore – unless explicitly stated otherwise – in what follows we use the term "serial entrepreneur" to indicate a portfolio business owners.

Practically, to identify serial owners in our data we count the number of businesses for which an individual is recorded as an owner in every year of our sample.[5] Next, we

[4]Worker level data are not available for the years of 1990 and 2001 and, therefore, we interpolate firm-level values for these years.

[5]As is typical in the literature, when measuring worker characteristics we restrict our sample to individuals aged 16 to 70. Fewer than 1% of all owners fall outside these bounds.

define an individual to be a (portfolio) serial entrepreneur if he or she simultaneously owns more than one business at *any* moment within our sample. It is important to note that under this definition, serial entrepreneurship is viewed as a "permanent characteristic" (fixed effect) of individuals. As will become clear, this definition of serial ownership will be particularly useful for our analysis of potential channels in Section 5. Nevertheless, the Appendix shows that our results are similar when we use a time-varying definition that categorizes individuals as serial owners only for those years in which they own multiple firms.

"Other" Entrepreneurs. We define "other" entrepreneurs as business owners who complement serial entrepreneurs, that is all business owners who are not classified as (portfolio) serial entrepreneurs. Therefore, as explained above, this definition of other business owners also includes sequential serial owners. The Appendix documents that the business performance of sequential owners is in fact very close to that of firms owned by individuals who only ever own one business (see also Shaw and Sørensen, 2019). This, therefore, explains our primary focus on portfolio owners only.

Firms of Other and Serial Entrepreneurs. Our primary units of observation are firms and we will use business and firm interchangeably throughout the paper. That said, certain parts of our analysis also make use of establishment-level information of the QP. When doing so, we will be explicit in distinguishing firms and establishments.

A key feature of our analysis is that we categorize firms by the characteristics of their owners. In particular, we classify businesses as "serial entrepreneur (SE) firms" if at any point in their life-cycles *at least one* of their owners is involved in multiple companies, i.e., classified as a serial business owner. All other businesses are classified as "other (O) firms". Note that $2/3$ of all firms in the Portuguese economy have only one owner. Nevertheless, the Appendix shows that our results are similar when we restrict our definition to firms which are *solely* owned by serial entrepreneurs.[6]

Owner-Managers. As already noted, our data allows us to distinguish owner-managers from owners without an active role in their businesses – e.g., because they are "only" investors in a given business. To identify owner-managers, we make use of information in the QP which classifies individuals into one of eight levels of job hierarchy based on tasks performed in their job and the skills required to do so. Specifically, we consider an owner to be also a manager if the owner is classified as one in the top level of hierarchy: "top executives (top management)".[7]

[6]While we observe whether an individual is an owner of a particular business, we do not have information on their ownership (equity) share.

[7]This classification is defined by Portuguese law (Lima and Telhado Pereira, 2003; Caliendo et al., 2020). See also Queiró (2022) for a related study of managers in Portugal.

The Appendix shows that the vast majority of business owners are also managers in Portugal. In particular, 93 percent of regular and 98 percent of serial entrepreneurs can be classified as owner-managers. Moreover, as we explain in detail in Section 5, whether or not a serial entrepreneur is also a manager does not seem to matter for the performance of their firms.

Discussion: Right Censoring, Selection on Survival and M&As. Having described the main concepts in this paper, we now discuss potential concerns arising from our specific definitions. The first two are common to all studies of serial entrepreneurship, while the third is specific to our macroeconomic focus.

First, our results may be biased because of right censoring. Specifically, business owners may be classified as "regular", despite the fact that they will start a second business in the future, *outside* our sample period. While this feature only likely creates a downward bias in estimates of the importance of serial owners (i.e., our results may be viewed as lower bounds), we provide an explicit robustness check using a truncated sample in the Appendix.

Second, studying serial entrepreneurship implies focusing on a select group of businesses. Intuitively, SE firms are more likely to display lower exit rates because first businesses of serial entrepreneurs need to survive before the owner starts another firm. Exceptions may be serial entrepreneurs who found multiple startups at the same time. Nevertheless, it remains an open question whether SE businesses contribute quantitatively to macroeconomic outcomes – precisely one of the questions addressed in this paper. Indeed, we view serial entrepreneurship as an *observable proxy* for underlying sources of firm performance, which we explicitly study in Section 5. Our approach is, therefore, analogous to the study of the macroeconomic impact of "high-growth" firms which relies on identifying such businesses with observed, realized, growth outcomes (see for example Haltiwanger et al., 2016).

Finally, to accurately quantify the macroeconomic contribution of SE firms, it is important to account for mergers and acquisitions (M&As). Intuitively, expansions (or entry) of SE firms via M&A activity should not be counted as contributions to aggregate job creation since they are exactly offset by exit of the acquired business.[8] However, M&As are not a quantitative concern in Portugal. Mata and Portugal (2004) report that M&As account for less than 1% of all business shutdowns. In addition, while M&A activity is not directly observable in our data, we provide an estimate by using worker tenure information. In particular, we consider a new firm to be the result of a merger or acquisition if any of its workers report having tenure larger than 1 year at the time

[8] In the data, the identification numbers of the firms involved in the M&A operation are transmitted to the resulting firm, while the others disappear. Therefore, the acquired businesses are counted as exits in the data.

of startup. Only about 3% of all startups in our sample would be classified as M&As according to this proxy.[9]

A.2 Outcome variables

Ultimately, our main units of observation are firms. To describe the performance of a group of firms, we focus on four distinct variables: size, growth, productivity and exit rates. We now explicitly define these outcome variables as well as our definition of high-growth firms, a key concept for studying business dynamism. Note that the majority of our results are based on the cross-section of firms. The exceptions are findings related to firms' life-cycle profiles, which report averages across *cohorts* of businesses.

Firm Size, Growth, Job Creation and Destruction. Because of the ease and quality of measurement, we focus on employment, E, as our baseline measure of firm size. This notion of firm size is consistent with a range of existing studies (see for example Moscarini and Postel-Vinay, 2012; Haltiwanger et al., 2013) and allows us to directly analyze the impact on aggregate job creation, often a key policy interest.

We follow Davis et al. (1996), henceforth DHS, and measure firm growth in firm i and period t, g_{it}, as

$$g_{it} = \frac{(E_{it} - E_{it-1})}{X_{it}}, \tag{A1}$$

where $X_{it} = 1/2(E_{it} + E_{it-1})$. Conveniently for our purposes, the DHS firm growth rate can be defined for different levels of aggregation:

$$g_t = \sum_s \frac{X_{st}}{X_t} g_{st} = \sum_s \left(\frac{X_{st}}{X_t} \sum_{i \in s} \left(\frac{X_{it}}{X_{st}} \right) g_{it} \right), \tag{A2}$$

where $X_t = \sum_s X_{st} = \sum_s \sum_{i \in s} X_{it}$ and where s denotes a particular group of firms – our primary focus will be on serial entrepreneur and other businesses, $s = \{SE, O\}$. The same concepts can also be used to define job creation (JCr) and destruction rates (JDr):

$$JCr_t = \sum_s \left(\frac{X_{st}}{X_t} \sum_{i \in s, g_{i,t} \geq 0} \left(\frac{X_{it}}{X_{st}} \right) g_{it} \right), \tag{A3}$$

$$JDr_t = \sum_s \left(\frac{X_{st}}{X_t} \sum_{i \in s, g_{i,t} < 0} \left(\frac{X_{it}}{X_{st}} \right) g_{it} \right). \tag{A4}$$

[9] Even when restricting attention to SE startups only, the fraction with workers reporting tenure larger than 1 year is about 6%. Therefore, while SE firms seem to be more often associated with M&A activity, the absolute number of such cases remains quantitatively small.

Productivity and Exit. Since accurate estimates of firm-level productivity are hard to obtain, we focus on the simplest measure of labor productivity $q_{i,t} = Z_{i,t}/E_{i,t}$, where $Z_{i,t}$ are sales of firm i in period t.[10]

Finally, in our analysis we also focus on firm exit rates, $D_{s,t}$. For a group of firms, s, We define the exit rate as the number of businesses which shut down in a given year relative to incumbent firms:

$$D_{s,t} = \frac{(\text{\# of exiting firms})_{s,t}}{(\text{\# of firms})_{s,t}}. \tag{A5}$$

As discussed above, exit rates of SE firms may be subject to a survival bias and, therefore, we interpret results pertaining to firm exit with caution.

High-Growth Firms ("Gazelles"). Part of our analysis focuses on high-growth firms, so called gazelles. We follow the Eurostat-OECD (see European Commission, 2007) definition of gazelles as businesses up to 5 years old, with a minimum of 10 employees (at some point in the firm's existence), and with average annualized growth of at least 20% per year, over a three year period. As with the definition of serial ownership, we treat the term gazelle as a permanent characteristic – a fixed effect – of a particular business. That is, once a young business satisfies the requirements to be classified as a high-growth firm, we continue to refer to such businesses as gazelles even beyond the age of 5 and even if their subsequent growth slows. This allows us to gauge how high-growth firms differ from other businesses throughout their life-cycles.

While informative for our purposes, other definitions of gazelles have been used in the literature. For example, Haltiwanger et al. (2016) define gazelles as businesses which exhibit an annual growth rate above 25% in any given year. This definition is not conditional on firm age, nor is it used as a "fixed effect." In the Appendix, we show that our results are similar when using this alternative definition of high-growth firms.

[10]While the QP includes revenue, it does not have information allowing us to estimate firm-level total factor productivity.

B Additional Empirical Results

This Appendix provides a range of robustness checks for our key empirical findings.

B.1 Alternative measurement of serial entrepreneurs

The results in Section 4 use the "fixed effect" definition of serial entrepreneurship where we treat any business owner who simultaneously owns multiple businesses as a serial entrepreneur *throughout the entire sample*. In this Appendix, we document that very similar results are obtained using the alternative, "year-by-year" definition, where we count as serial entrepreneurs only those business owners who own multiple businesses in a given year. This is intuitive, since in Section 5 we show that first and subsequent firms of serial entrepreneurs have similar characteristics.

Specifically, Table A1 and A2 document that even under the year-by-year definition portfolio serial entrepreneur (SE-P) firms still enjoy a significant premium over other businesses and that they disproportionately contribute to aggregate job creation and productivity growth. In addition, the following Figures show that under the alternative definition SE-P firms still have a wider size distribution which fans out faster with firm age (Figure A1), they shape average up-or-out dynamics (Figure A2) and that even the very first businesses of serial entrepreneurs outperform other businesses (Figure A3).[11]

Table A1: Serial entrepreneur premium: year-by-year definition

	Other	SE-P	SE-P Premium
Size (workers)	5.2	11.0	0.52^{***}
Exit (in %)	7.8	6.9	-0.45^{***}
Growth (in %)	9.6	10.6	2.13^{***}
Productivity (agg. = 1)	0.88	1.26	0.31^{***}

Notes: The columns show, respectively, the unconditional averages of other and portfolio serial entrepreneur firms and the SE-P premium, as estimated in the main text. The rows depict, respectively, average size (employment), exit rates, (employment-weighted) net employment growth and average labor productivity scaled by labor productivity of all firms. Ordinary Least Squares estimates with robust standard errors clustered at the firm level in parentheses. *** stands for statistical significance at the 1% level. Serial entrepreneur businesses are defined using the year-by-year definition.

[11] Note that exit rates of SE and R firms are much closer to each other, *unconditionally*. Nevertheless, estimating the SE exit premium still reveals that even year-on-year serial entrepreneur firms exit less frequently than regular businesses.

Table A2: Contributions to aggregates (in %): year-by-year definition

	Firms	Employment	Job creation	Job destruction	Productivity growth
Other	95.3	90.6	91.2	92.2	95.2
SE-P	4.7	9.4	8.8	7.8	4.8

Notes: The table shows, respectively, the contributions (in %) of "other' and "SE-P" businesses to the aggregate number of firms, employment, job creation, job destruction and productivity growth.

Figure A1: Firm size distribution and evolution: year-by-year definition

(a) Overall firm size distribution

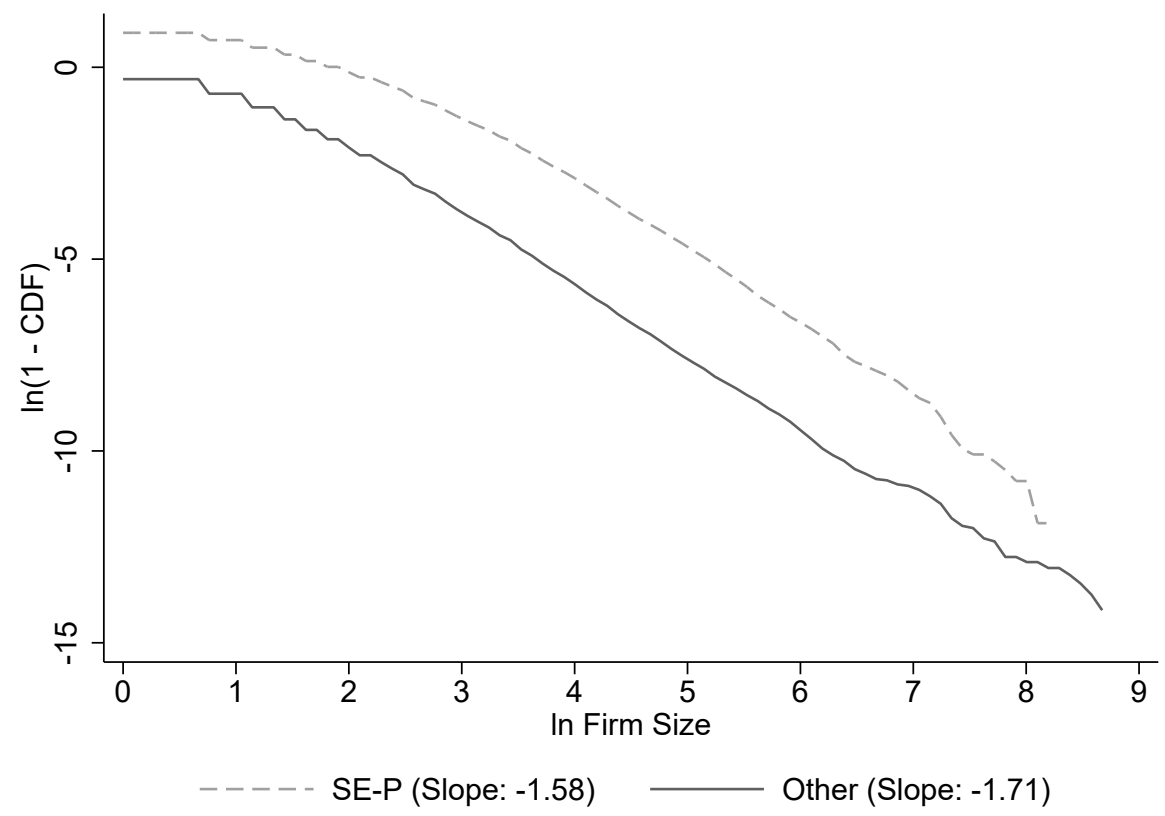

(b) Evolution of firm size distribution

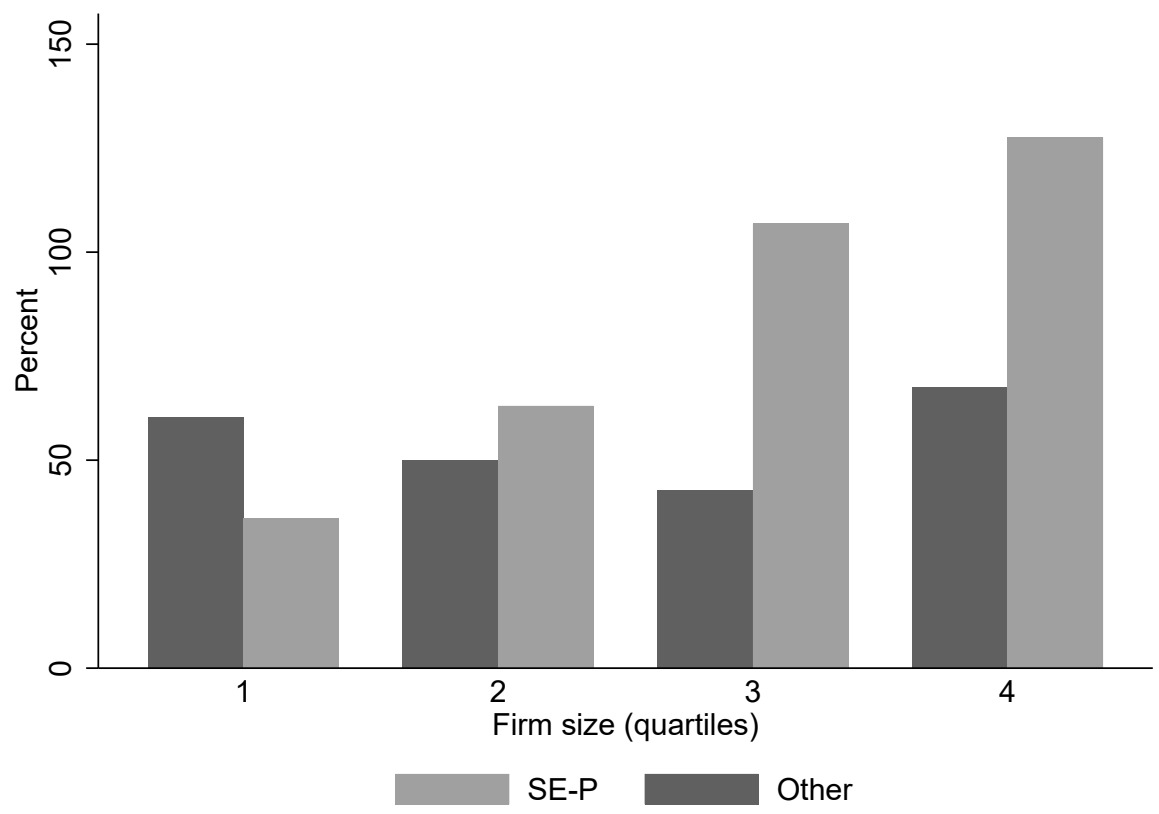

Notes: Panel (a) of the figure shows the firm size distribution of other and SE-P firms and Panel (b) visualizes the respective evolution by plotting the size growth (between startups and 10 year old firms) in the quartiles of the respective distributions. Portfolio serial entrepreneur businesses are defined using the year-by-year definition.

Figure A2: Size and exit profiles by age: year-by-year definition

(a) Firm size

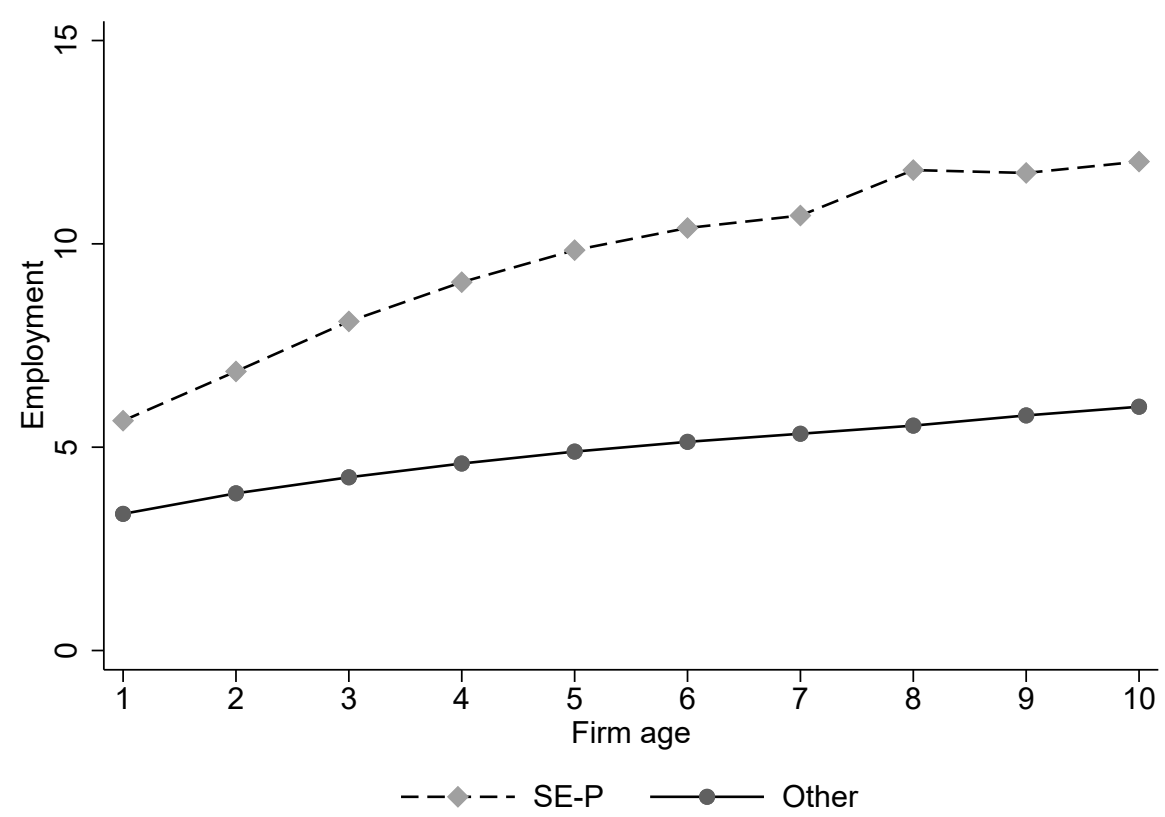

(b) Exit rates

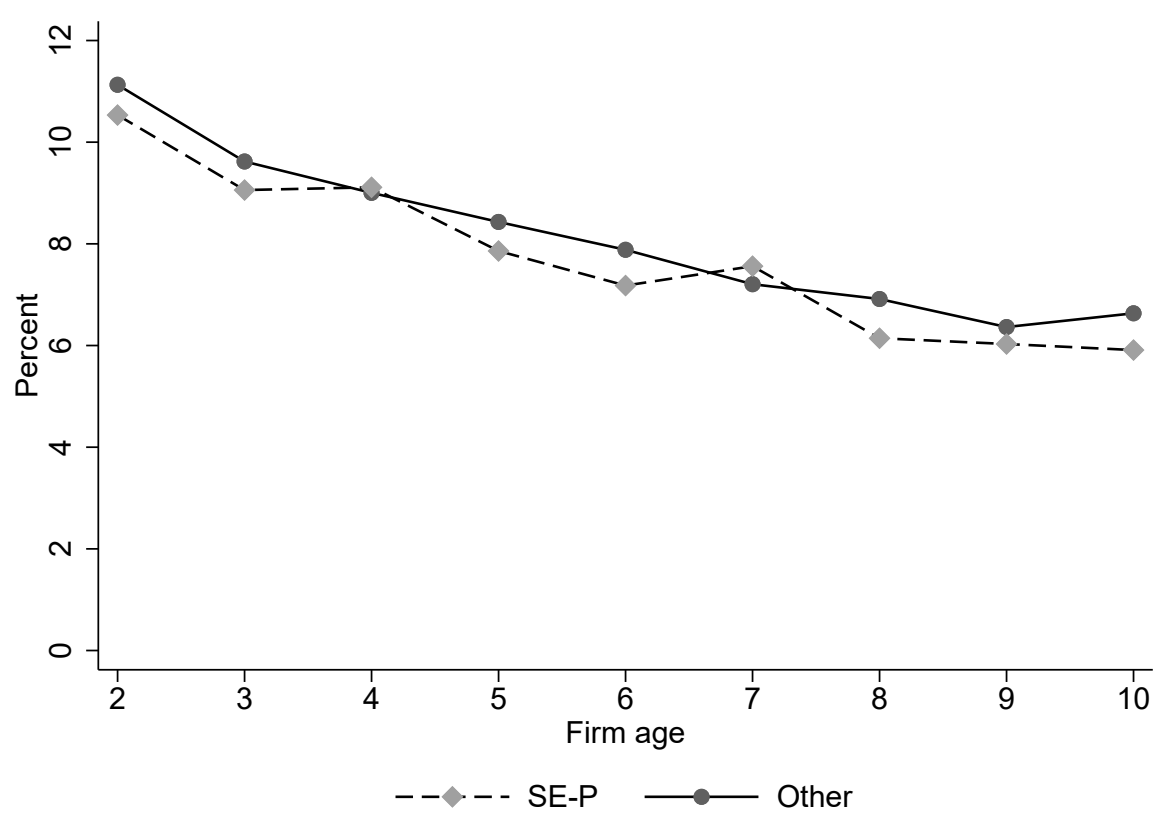

Notes: The left panel shows average firm size by age, while the right panel shows average exit rates by age. Both panels depict other and SE-P businesses. Portfolio serial entrepreneur businesses are defined using the year-by-year definition.

Figure A3: Size and exit profiles by age: First SE, Subsequent SE and Other firms: year-by-year definition

(a) Firm size

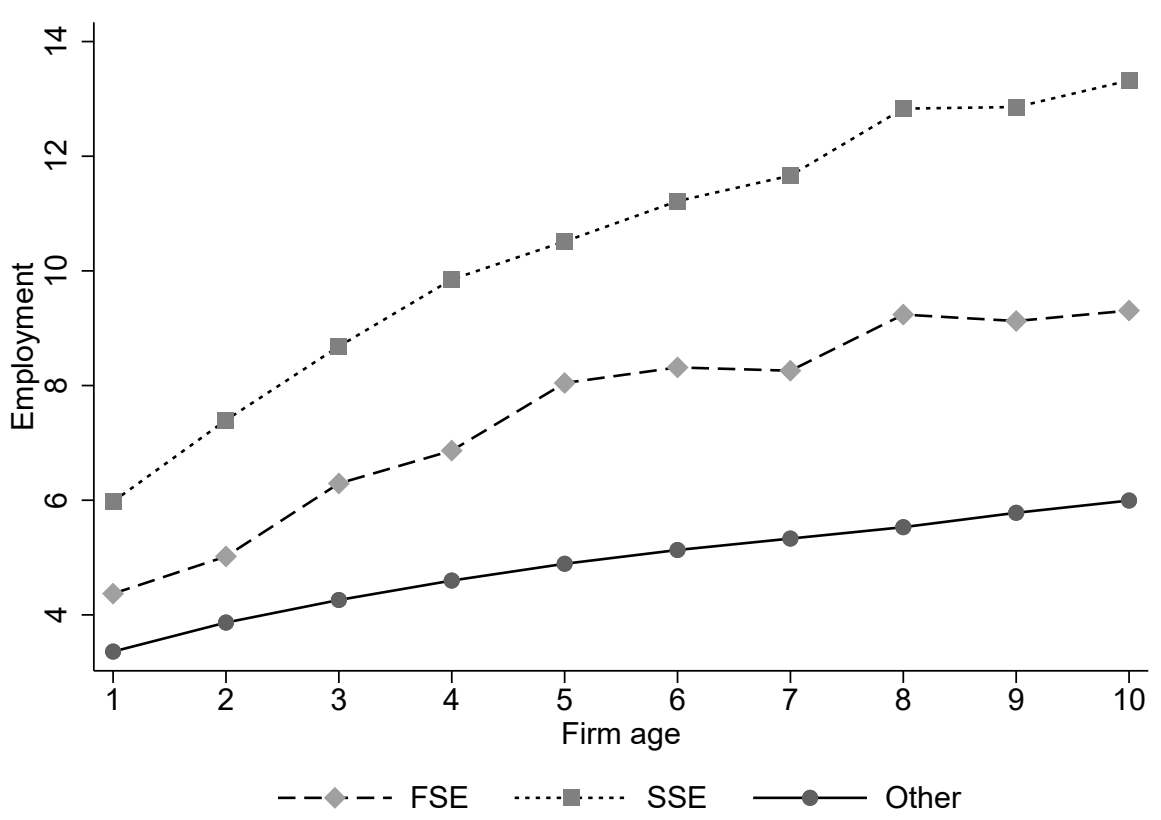

(b) Firm exit

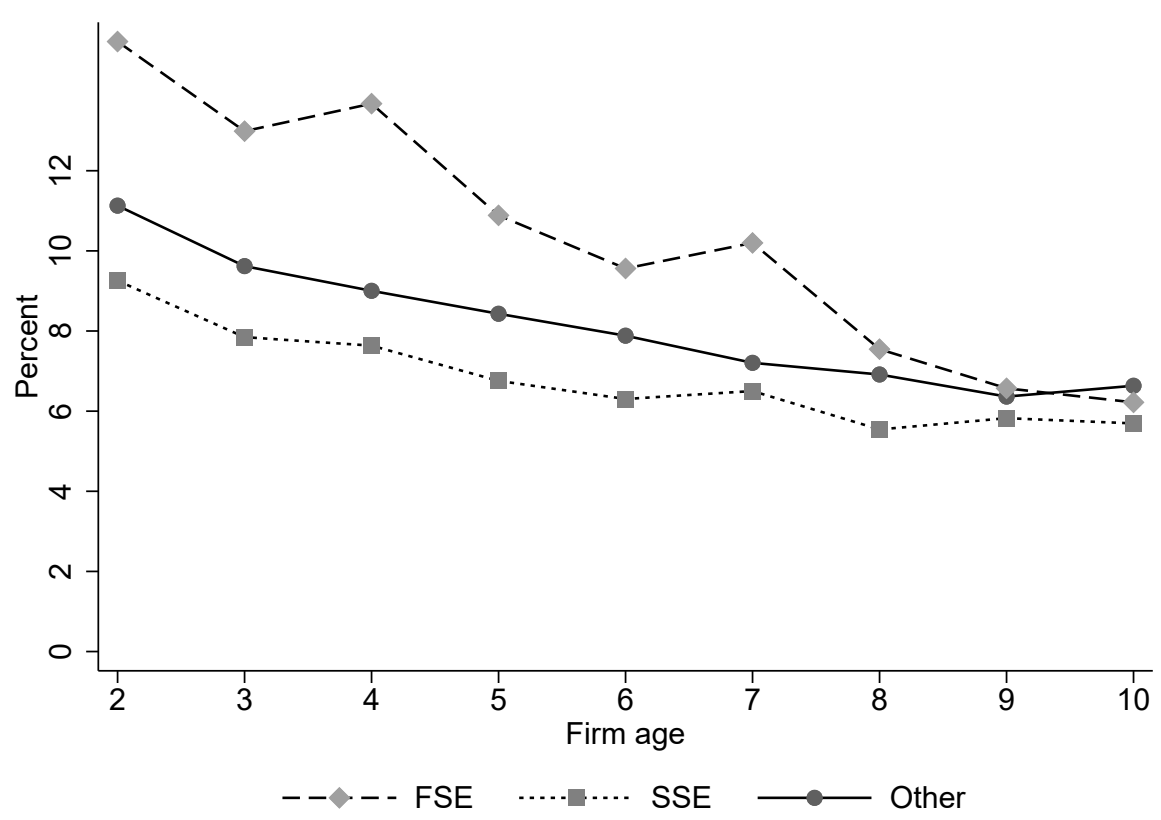

Notes: The left panel shows average firm size by firm age, while the right panel shows average exit rates by firm age. Both panels depict other and portfolio serial entrepreneur businesses, where the latter are split into first and subsequent businesses. Portfolio serial entrepreneur businesses are defined using the year-by-year definition.

B.2 Sequential vs Portfolio Entrepreneurs

The main text defines serial entrepreneurs as business owners who *simultaneously* own multiple firms. These entrepreneurs have been dubbed "portfolio" serial entrepreneurs in the literature. In this Appendix, we show that businesses of "sequential" serial entrepreneurs, those who own multiple businesses with a non-entrepreneur spell in between (i.e., their first business shuts down before they start another), are more similar to firms that are always owned by a single owner, referred to as novice (N) businesses. Precisely for this reason we focus on portfolio serial entrepreneurs in the main text.

Figure A4: Size and exit profiles by age: Novice, portfolio and sequential businesses

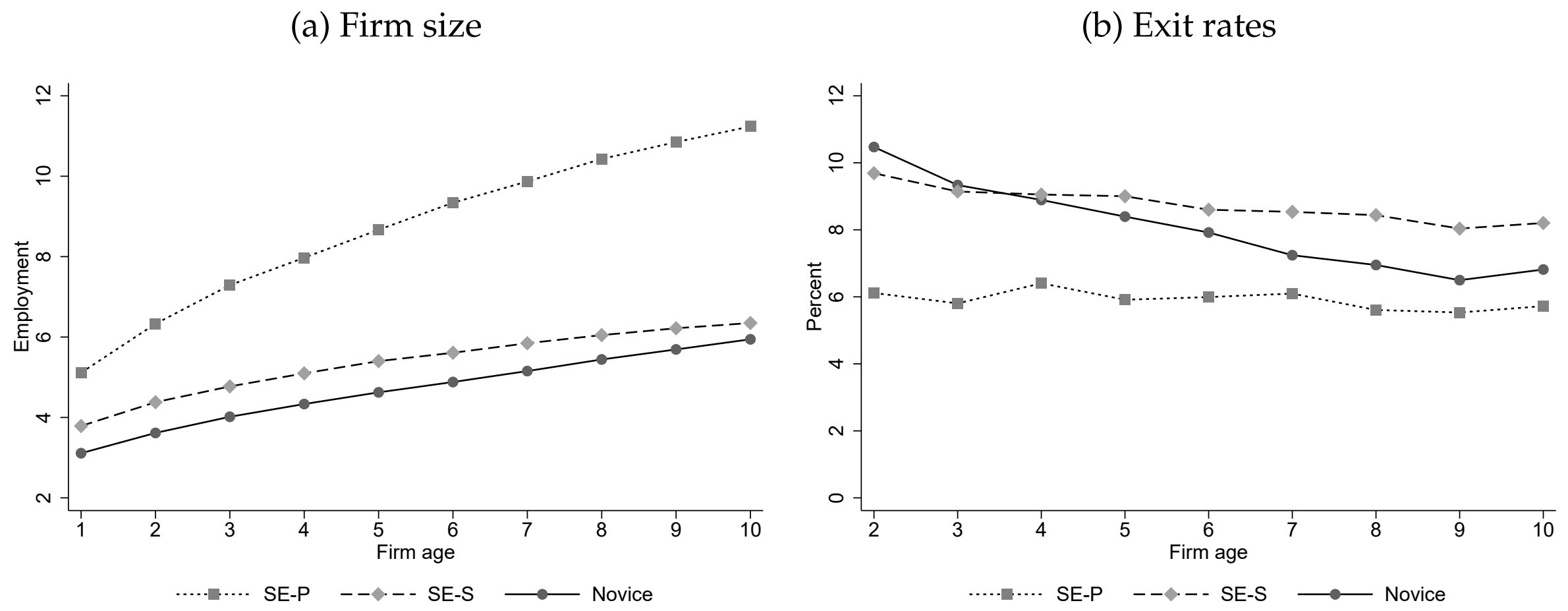

Notes: The left panel shows average firm size by age, while the right panel shows average exit rates by age. Both panels depict portfolio serial, sequential serial and novice businesses.

Specifically, Figure A4 shows that firm performance of sequential serial entrepreneurs is very close to that of the group of novice entrepreneurs. The only noticeable difference seems to be that firms of sequential entrepreneurs start (and remain) larger on average, compared to those of novice business owners.

Table A3 presents estimates of premia associated with sequential serial entrepreneurs. The table confirms that sequential serial entrepreneurs own larger businesses compared to novice entrepreneurs. However, neither of the other characteristics (exit rates, growth or productivity) display a superior premium.

Table A3: SE premia over novice, portfolio and sequential entrepreneurs

	N	SE-S	SE-P	Premia	
				(SE-S)-N	(SE-P)-(SE-S)
Size (workers)	5.3	5.8	10.9	0.19***	0.35***
Exit (in %)	7.9	8.2	5.4	0.64***	-2.49***
Growth (in %)	8.9	8.8	9.8	-0.35*	2.18***
Productivity (agg.=1)	0.79	0.88	1.17	0.00	0.26***

Notes: The first three columns show, respectively, the averages of novice, sequential and portfolio serial entrepreneur firms. Columns 4 and 5 show the estimated premia. "(SE-S)-N" is the premium of sequential entrepreneur businesses over novice firms and "(SE-P)-(SE-S)" is the premium of portfolio over sequential serial entrepreneur firms. The rows depict, respectively, average size (employment), exit rates, (employment-weighted) size growth and firm-level labor productivity scaled by labor productivity of all businesses. Ordinary Least Squares estimates with robust standard errors clustered at the firm level in parentheses. *** and * stand for, respectively, statistical significance at the 1% and 10% levels.

B.3 Analysis on a truncated sample period

As is typical in studies of serial entrepreneurship, also our analysis suffers from a right censoring bias. In particular, we categorize as regular entrepreneurs even those business owners who will become serial entrepreneurs in the future, *outside our sample*. While this bias likely leads to our results being a lower bound (since some of our regular entrepreneurs are in fact future serial entrepreneurs which – as our results suggest – own businesses with superior performance), this Appendix provides results where we explicitly address the bias.

Towards this end, we redo our analysis on a truncated sample. Specifically, we truncate the end of our sample in 2009 instead of letting it end in 2017. This truncation closely mimics the average number of years it takes a serial entrepreneur to start their second business. The truncation results in "reclassifying" 12.6% of serial entrepreneurs in our baseline specification as other entrepreneurs because they start their subsequent businesses after 2009. Nevertheless, Table A4 and the following Figures show that are key results hold even for this truncated sample.[12]

Table A4: Contributions to aggregates (in %): truncated sample

	Firms	Employment	Job creation	Job destruction	Productivity growth
Other	87.1	78.9	79.9	83.2	80.8
Serial	12.9	21.1	20.1	16.8	19.2

Notes: The table shows, respectively, the contributions (in %) of "other" and "serial entrepreneur" businesses to the aggregate number of firms, employment, job creation, job destruction and productivity growth. Estimates are based on a truncated sample, ending in 2009 instead of 2017.

[12]The firm size distribution seems to fan out slightly less quickly, compared to the full sample. Nevertheless, average firm size of SE firms still grows faster on average, compared to R firms even in the truncated sample.

Figure A5: Size and exit profiles by age: truncated sample

(a) Firm size (b) Exit rates

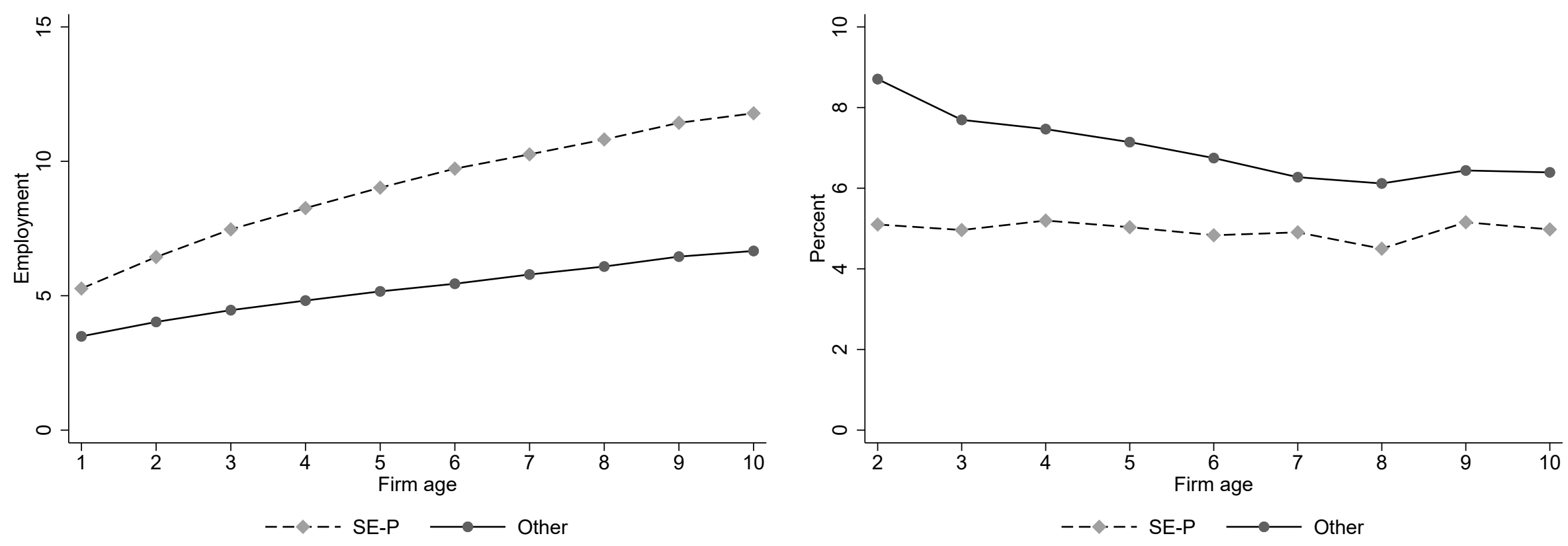

Notes: The left panel shows average firm size by age, while the right panel shows average exit rates by age. Both panels depict other and serial entrepreneur businesses. Estimates are based on a truncated sample, ending in 2009 instead of 2017.

Figure A6: Size and exit profiles by age: Other, First SE and Subsequent SE firms: truncated sample

(a) Firm size (b) Firm exit

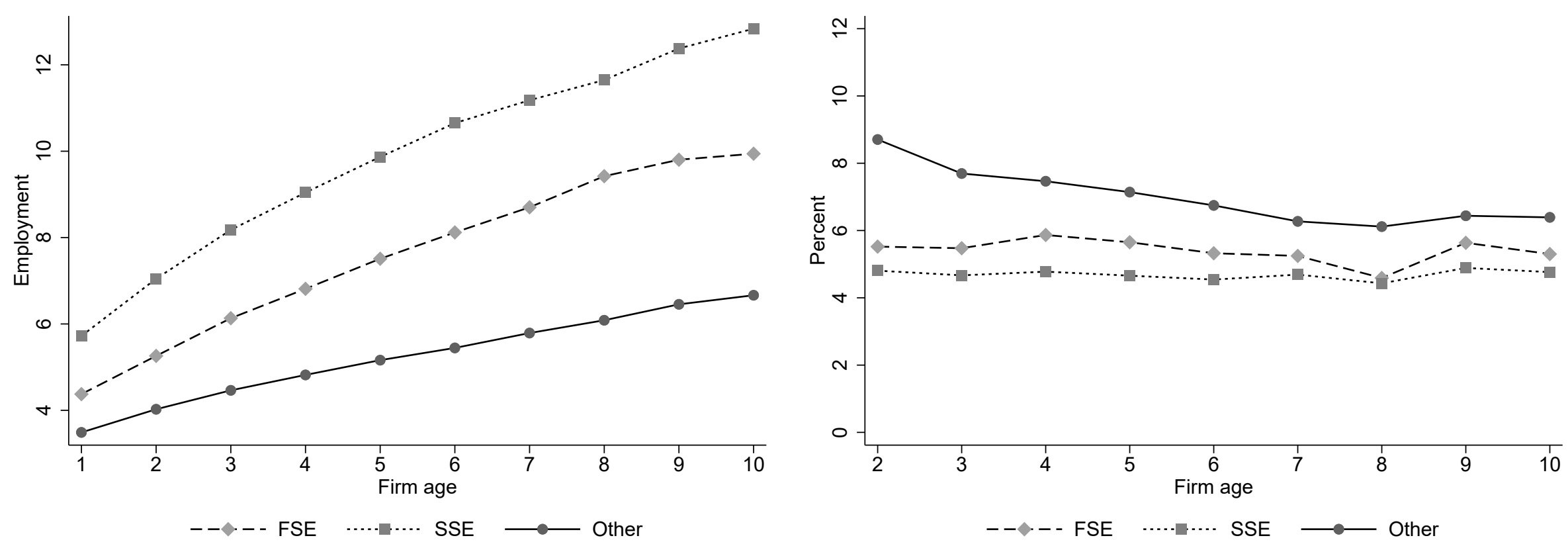

Notes: The left panel shows average firm size by firm age, while the right panel shows average exit rates by firm age. Both panels depict other and serial entrepreneur businesses, where the latter are split into first and subsequent businesses of serial entrepreneurs. Estimates are based on a truncated sample, ending in 2009 instead of 2017.

Figure A7: Firm size distribution and evolution: truncated sample

(a) Overall firm size distribution

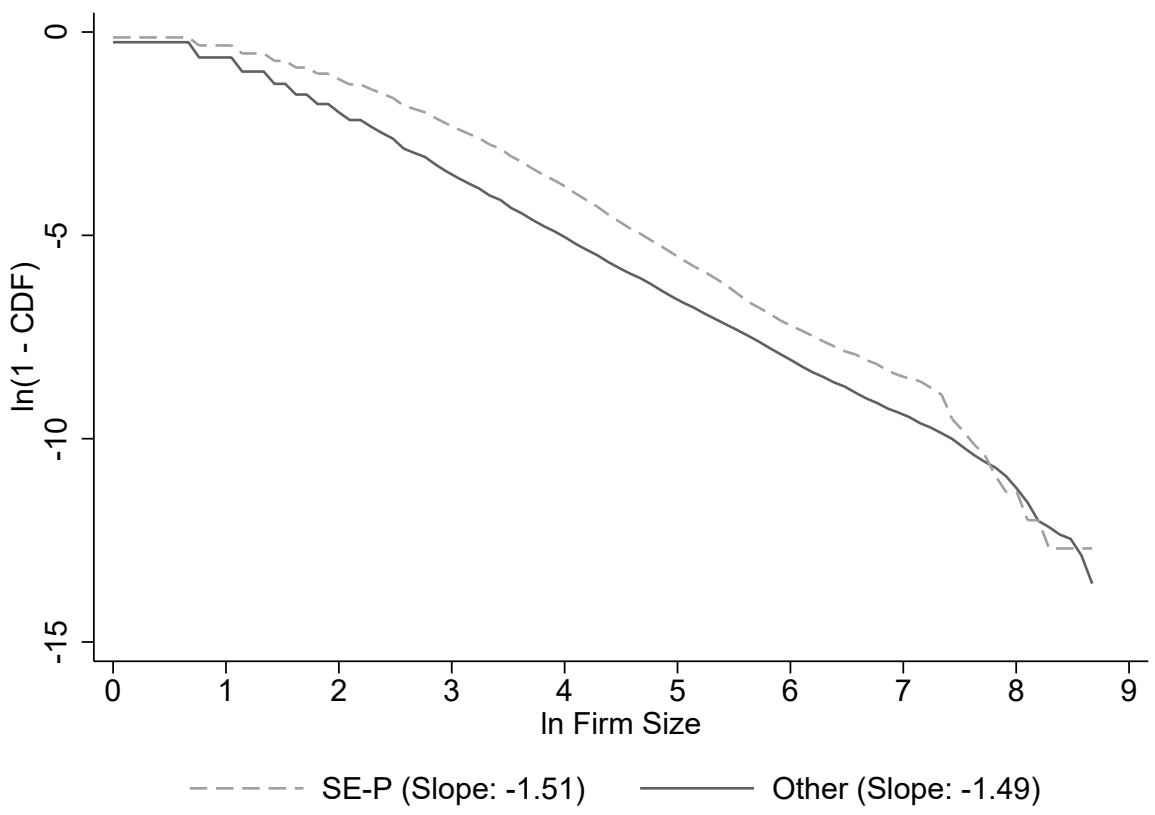

(b) Evolution of firm size distribution

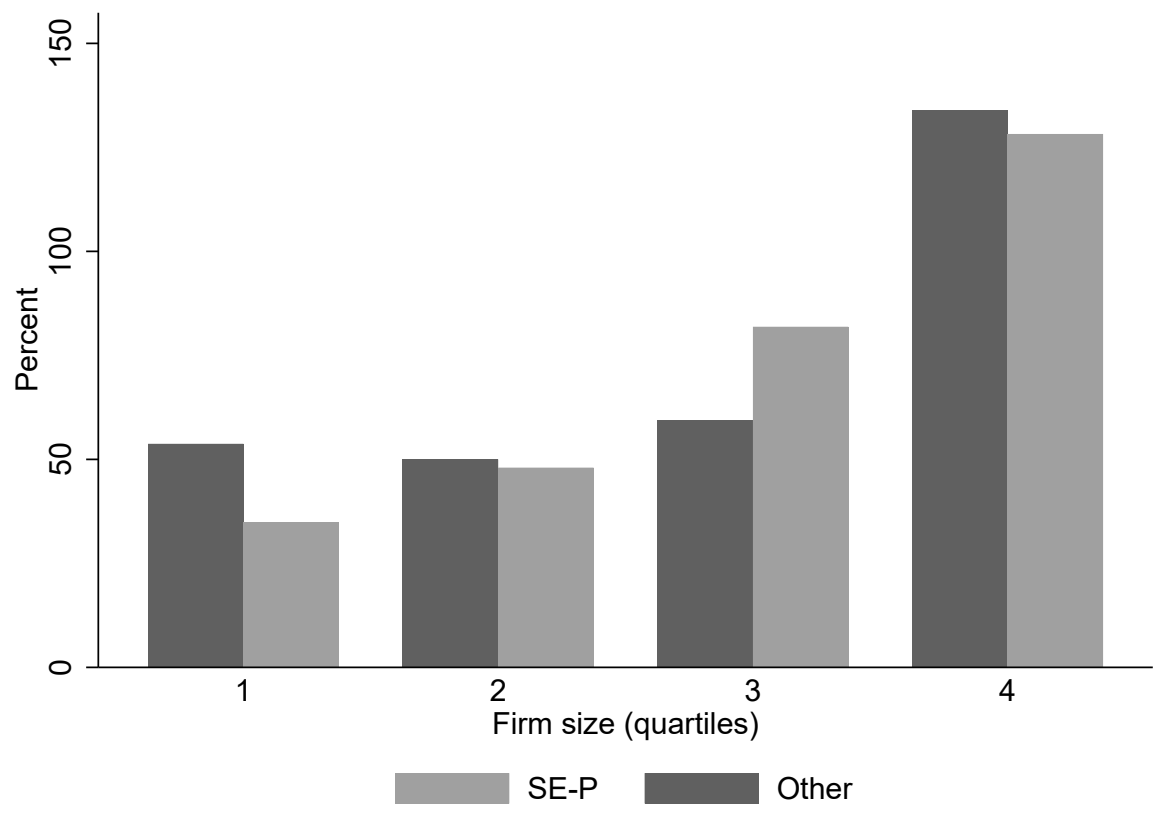

Notes: Panel (a) of the figure shows the firm size distribution of other and serial entrepreneur firms and Panel (b) visualizes the respective evolution by plotting the size growth (between startups and 10 year old firms) in the quartiles of the respective distributions. Estimates are based on a truncated sample, ending in 2009 instead of 2017.

B.4 Sole ownership only

In the main text, we classify serial entrepreneur firms as any business for which at least one owner is a serial entrepreneur. In this Appendix, we refine this definition to focus only on those businesses which have only a *single* owner. Those with a single serial entrepreneur owner are classified as SE-P firms, while all other businesses (i.e., including firms with multiple owners – serial entrepreneurs or not) are classified as "other". Table A5 and Figures A8 to A10 show that our key results remain to hold, albeit to a smaller quantitative extent (especially the firm-size distribution is quite close between O and SE-P firms). This is not surprising as the superior performance of part-owned SE firms (which make up almost 60% of all, solely and partly-owned, SE firms) is counted towards O businesses. Nevertheless, even then, solely owned SE-P firms are larger, growth faster and disproportionately contribute to aggregate job creation and productivity growth.

Table A5: Contributions to aggregates (in %): sole serial entrepreneurship

	Firms	Employment	Job creation	Job destruction	Productivity growth
Other	94.1	92.6	92.4	93.0	90.1
SE-P	5.9	7.4	7.6	7.0	9.9

Notes: The table shows, respectively, the contributions (in %) of "other" and solely owned"SE-P" businesses to the aggregate number of firms, employment, job creation, job destruction and productivity growth. In contrast to the main text, SE-P firms in this Appendix are restricted to have only a single owner.

Figure A8: Firm size distribution and evolution: sole serial entrepreneurship

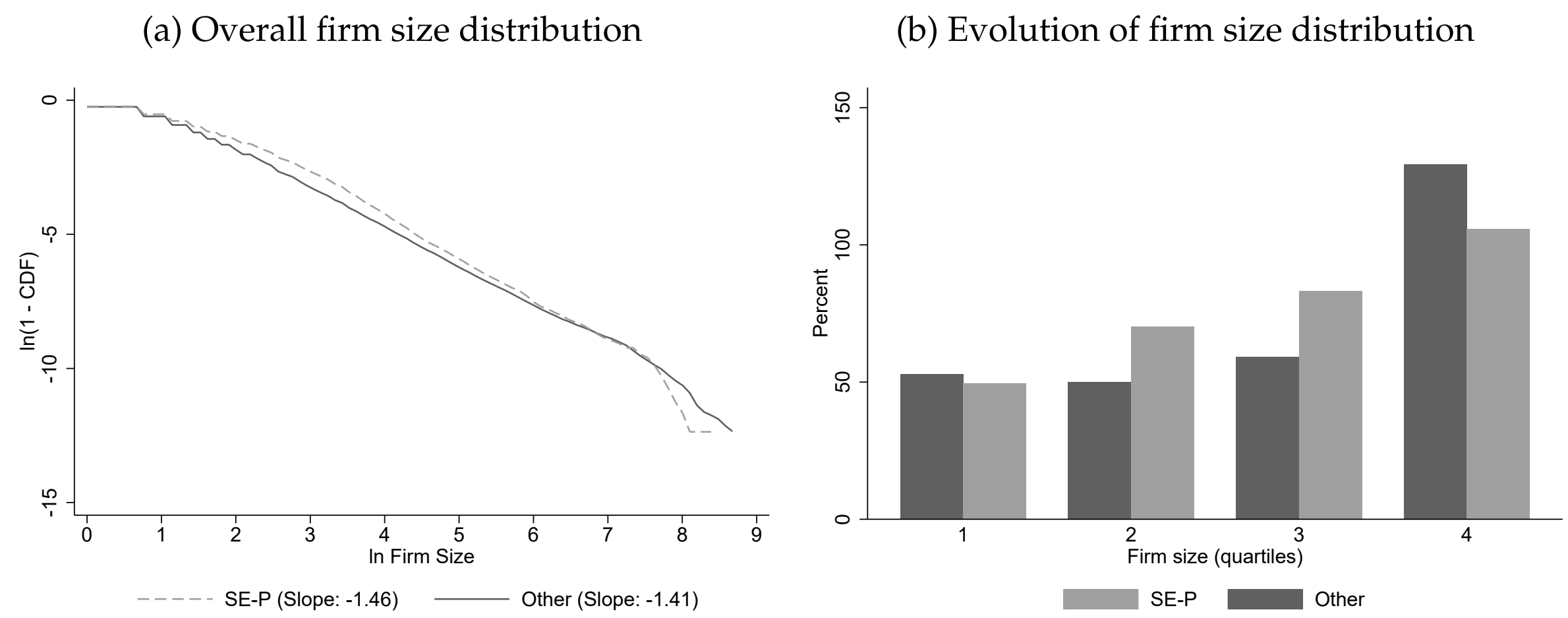

Notes: Panel (a) of the figure shows the firm size distribution of serial entrepreneur and other firms and Panel (b) visualizes the respective evolution by plotting the size growth (between startups and 10 year old firms) in the quartiles of the respective distributions. In contrast to the main text, SE-P firms in this Appendix are restricted to have only a single owner.

Figure A9: Size and exit profiles by age: sole serial entrepreneurship

(a) Firm size

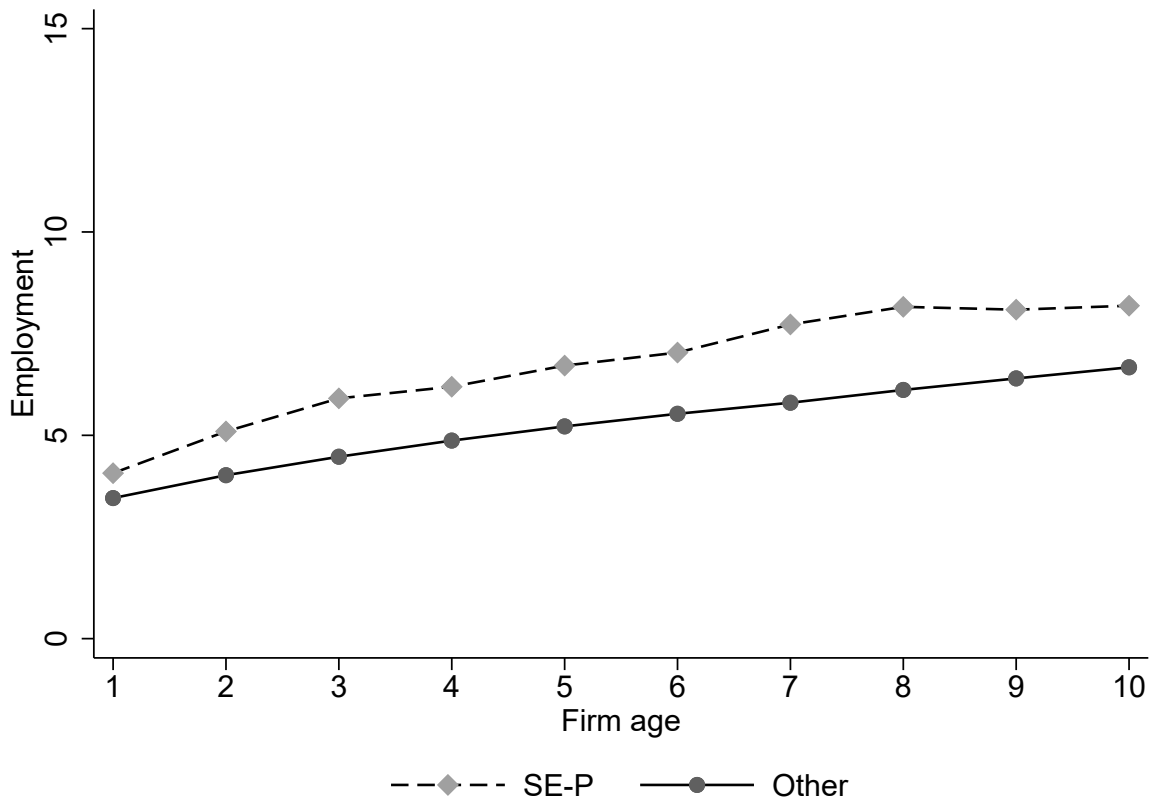

(b) Exit rates

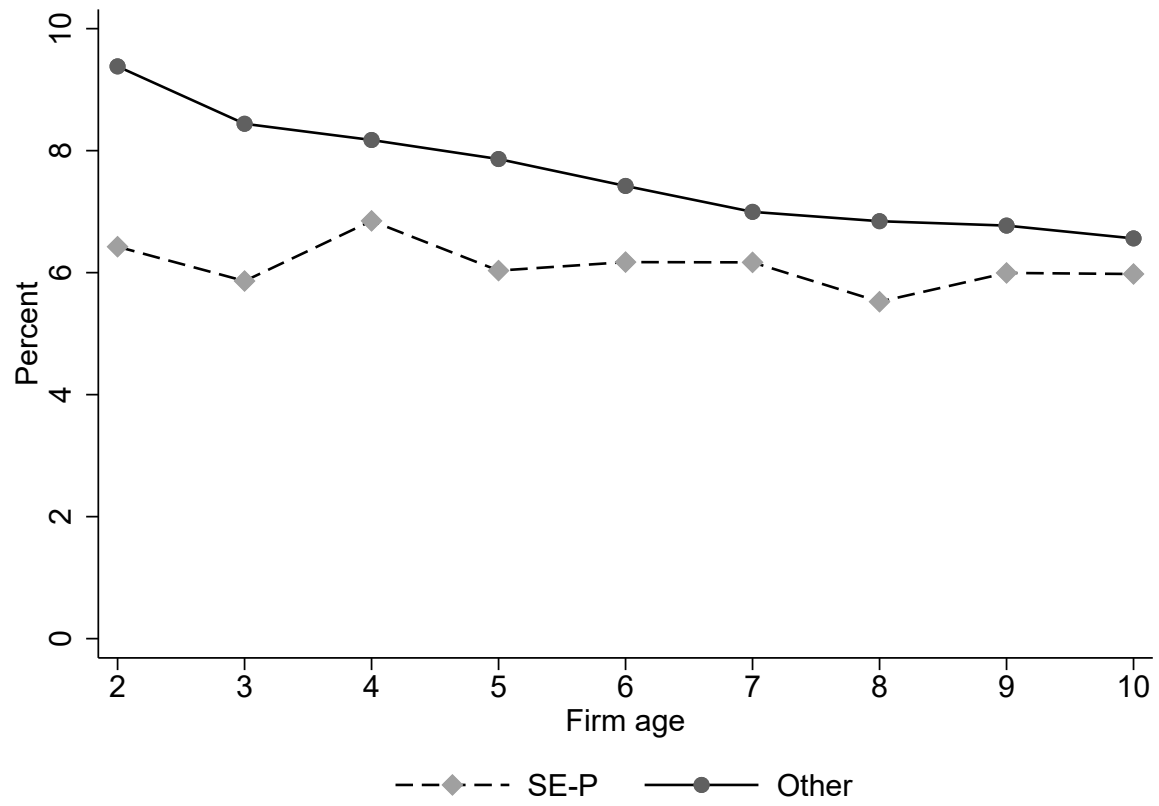

Notes: The left panel shows average firm size by age, while the right panel shows average exit rates by age. In contrast to the main text, SE-P firms in this Appendix are restricted to have only a single owner.

Figure A10: Size and exit profiles by age: Other, First SE and Subsequent SE firms: sole serial entrepreneurship

(a) Firm size

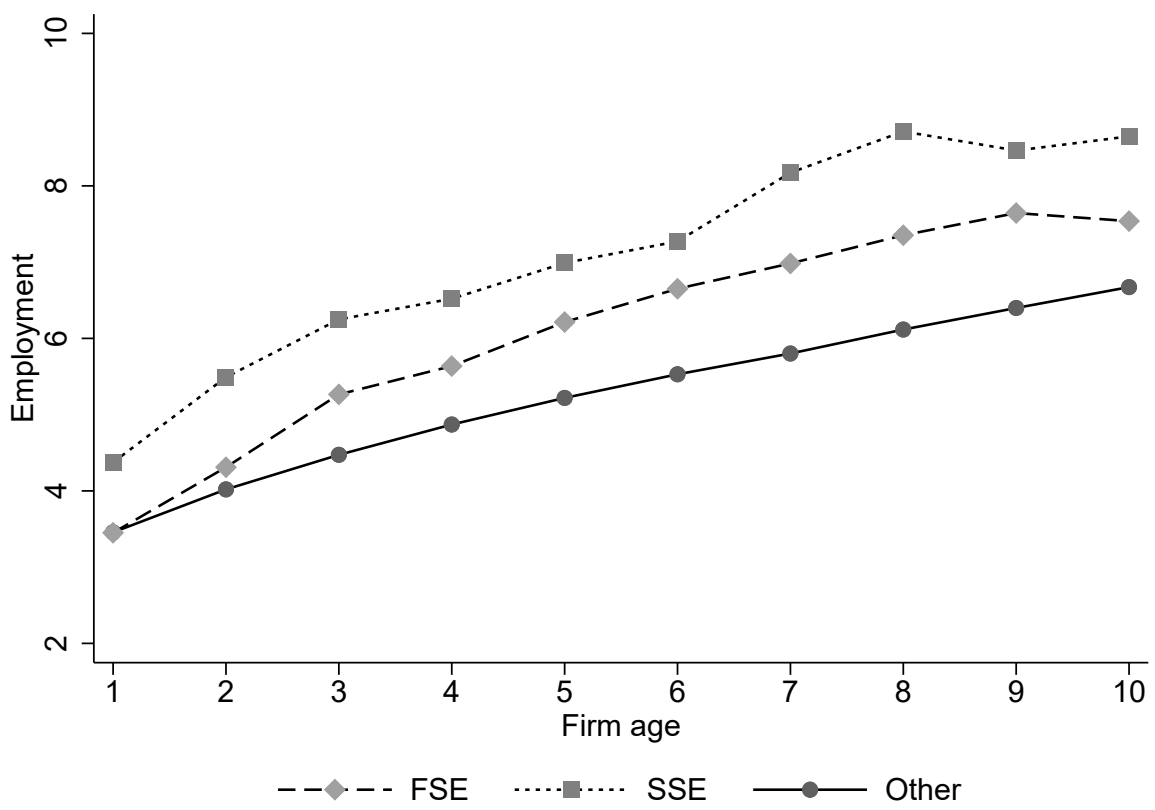

(b) Firm exit

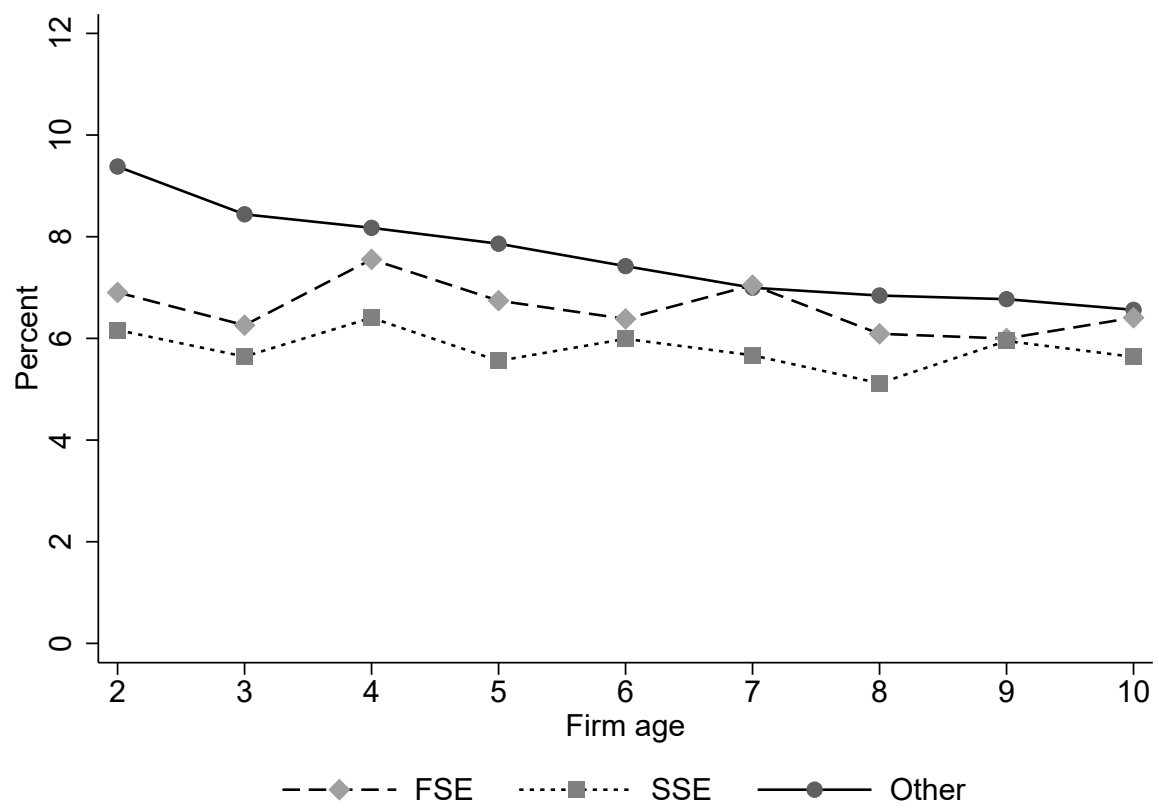

Notes: The left panel shows average firm size by firm age, while the right panel shows average exit rates by firm age. Both panels depict other and serial entrepreneur businesses, where the latter are split into first and subsequent businesses. In contrast to the main text, SE-P firms in this Appendix are restricted to have only a single owner.

B.5 Alternative definition of high-growth firms

The main text defined gazelles following the Eurostat-OECD definition (see European Commission, 2007). In this Appendix, we consider an alternative definition of gazelles. In particular, we follow Haltiwanger et al. (2016) and define gazelles as firms with annual growth rates higher than 25%. Note that this definition does not condition on firm age, nor does it consider gazelles to be a permanent characteristics as we is assumed in the main text.

Tables A6 and A7 show that even under an alternative definition of gazelles, high-growth firms still remain to be disproportionately important for aggregate employment and job creation and gazelles owned by serial entrepreneurs outperform other high-growth firms.

Table A6: Contribution of high-growth firms to aggregates (in %): alternative definition

	All gazelles	SE gazelles
Firms	19.3	15.4
Employment	22.2	24.3
Job creation	73.5	24.2

Notes: The table reports characteristics of all continuing high-growth firms (HW firm-year definition: employment growth above 25%) (first column) and those owned by serial entrepreneurs (second column). Shares are in % of all businesses in the first column, while they are a fraction of all high-growth firms in the second column.

Table A7: Serial Entrepreneur Premium for high-growth firms: alternative definition

	Other	Serial	SE Premium
Size (workers)	6.8	12.3	0.361***
Growth (in %)	58.3	59.2	1.56*
Productivity (aggregate = 1)	0.88	1.25	0.198***

Notes: The columns show, respectively, the averages of other and serial entrepreneur continuing high-growth firms and the estimated SE premium. The rows depict, respectively, average size (employment), job creation rates. Ordinary Least Squares estimates with robust standard errors clustered at the firm level in parentheses. *** stands for statistical significance at 1%.

B.6 Productivity Decomposition Among Continuing Firms Only

While the main text considered a decomposition of aggregate productivity growth into the contributions of incumbent as well as entering and exiting firms, this Appendix focuses only on the former. In particular, even only within incumbent businesses, SE firms contribute disproportionately to aggregate productivity growth which is especially true for the "within" component of growth.

Table A8: Aggregate productivity growth decomposition

	Total	Within	Between	Cross
Aggregate	6.5	10.5	3.7	−7.7
Serial entrepreneur firms: level	1.1	2.5	0.4	−1.8
Serial entrepreneur firms: share of aggregate	16.9	23.8	10.8	23.4

Notes: The table reports values (in %) from the productivity growth decomposition for continuing firms only. The first row reports aggregates, the second and third rows reports the contribution of serial entrepreneur firms only in levels and as a share of the aggregate, respectively.

B.7 Controlling for Industry and Location

In the main text, we focus on "raw" averages when analyzing firms' life-cycle profiles. While our results suggest that serial entrepreneurship is not a feature of a few particular industries, in this Appendix we control for industry and geographical location explicitly.

In particular, we estimate the following regressions

$$y_{j,t} = \alpha + \sum_{a} \delta_a \mathbb{1}(a) + \sum_{i} \delta_i \mathbb{1}(i) + \sum_{g} \delta_g \mathbb{1}(g) + \epsilon_{j,t}, \tag{A6}$$

where $y_{j,t}$ is a variable of interest (size or exit rates) of firm j in period t, a, i and g indicate, respectively firm age, industry and geographical location of operation, $\mathbb{1}$ are the associated indicator functions and the δ's are the respective estimated coefficients. We estimate the above for the three groups of firms, namely serial entrepreneur (SE-P) firms, other firms and all firms separately.

Figures A11 and A12 plot the estimated size and exit life-cycle profiles. For a given age a and particular firm type (SE-P, other or all), the latter is given by $\alpha + \delta_a$. The results suggest that controlling for industry and geographical location changes little on our results. The only exception is the initial size of SE-P firms which is now very similar to that of other businesses. However, the considerably stronger growth rate of SE-P firms remains apparent, even when controlling for industry and location effects.

Figure A11: Size and exit profiles by age

(a) Firm size (b) Exit rates

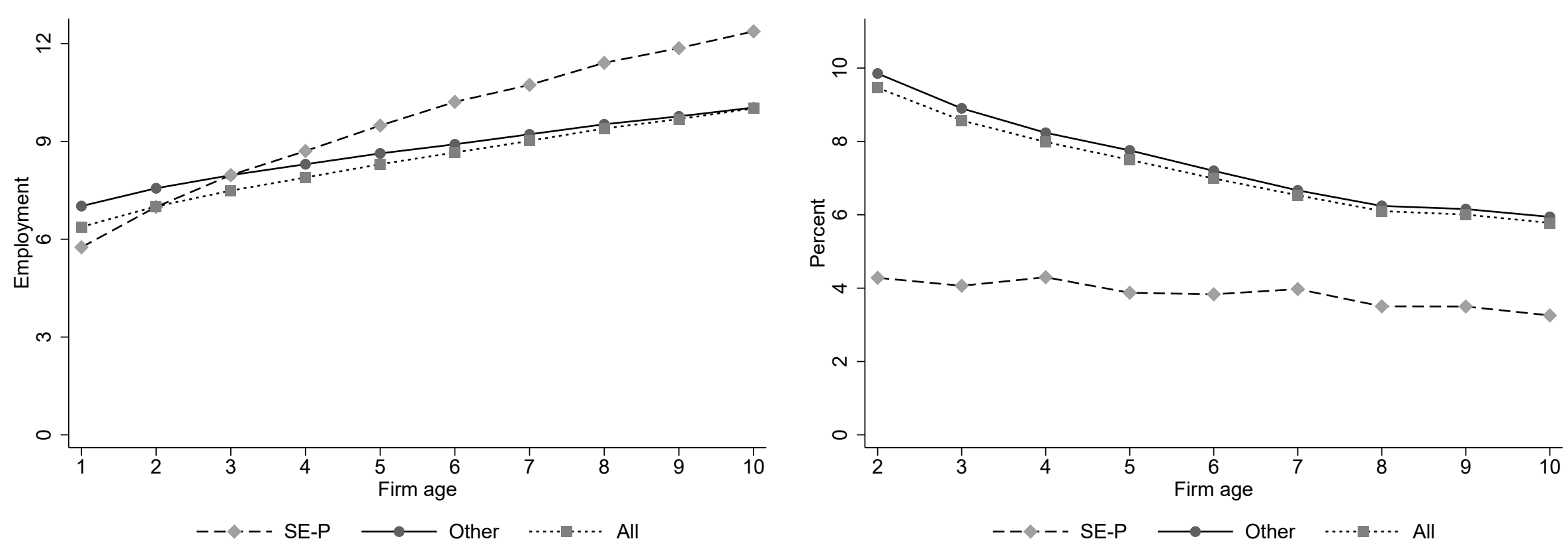

Notes: The left panel shows average firm size by age, while the right panel shows average exit rates by age. Both panels depict serial entrepreneur, other and all businesses.

Figure A12: High-growth firms: Size and exit profiles by age

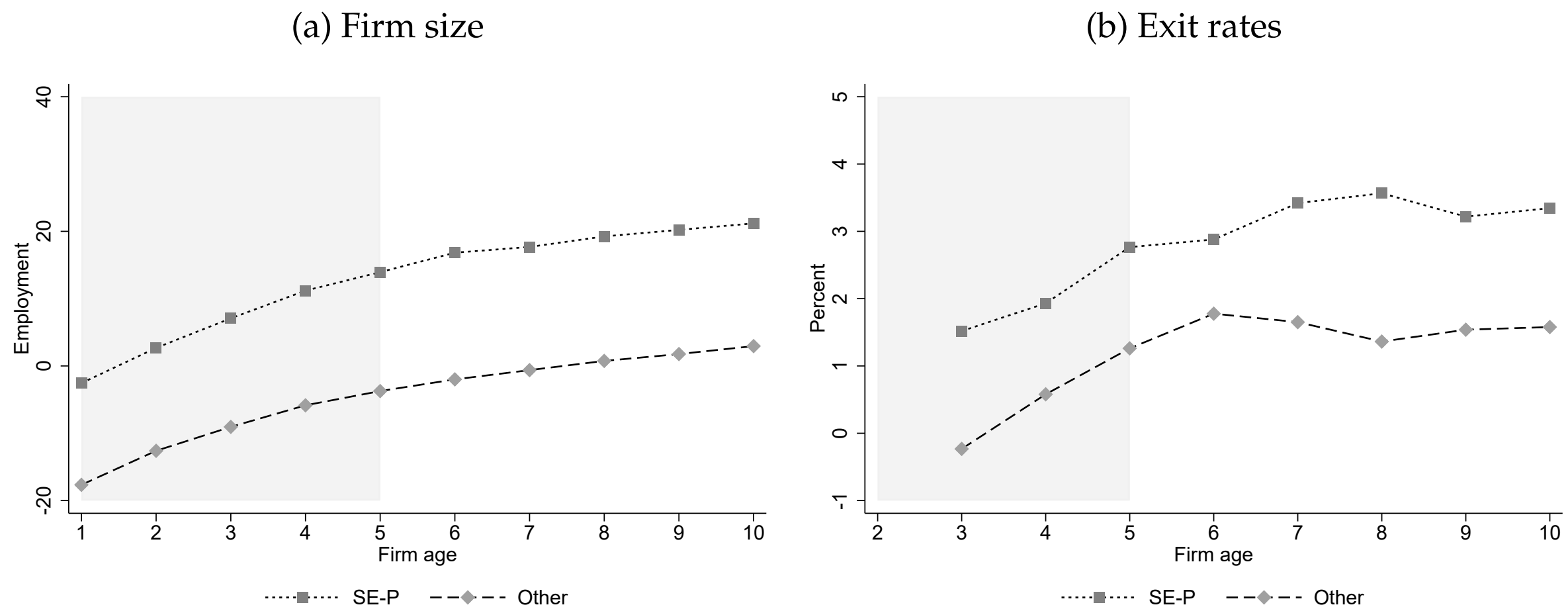

Notes: The left panel shows average firm size by age, while the right panel shows average exit rates by age. Both panels depict SE-P and other entrepreneur owned high-growth businesses. We define gazelles as businesses younger than five years which display an annual growth rate of at least 20 percent for at least 3 years and employ at least 10 workers at some point in their life-time (see European Commission, 2007). Shaded areas indicate the five year period used for defining high-growth firms.

Figure A13: Margins of growth

(a) Size of single-establishment firms

(b) Establishments per firm

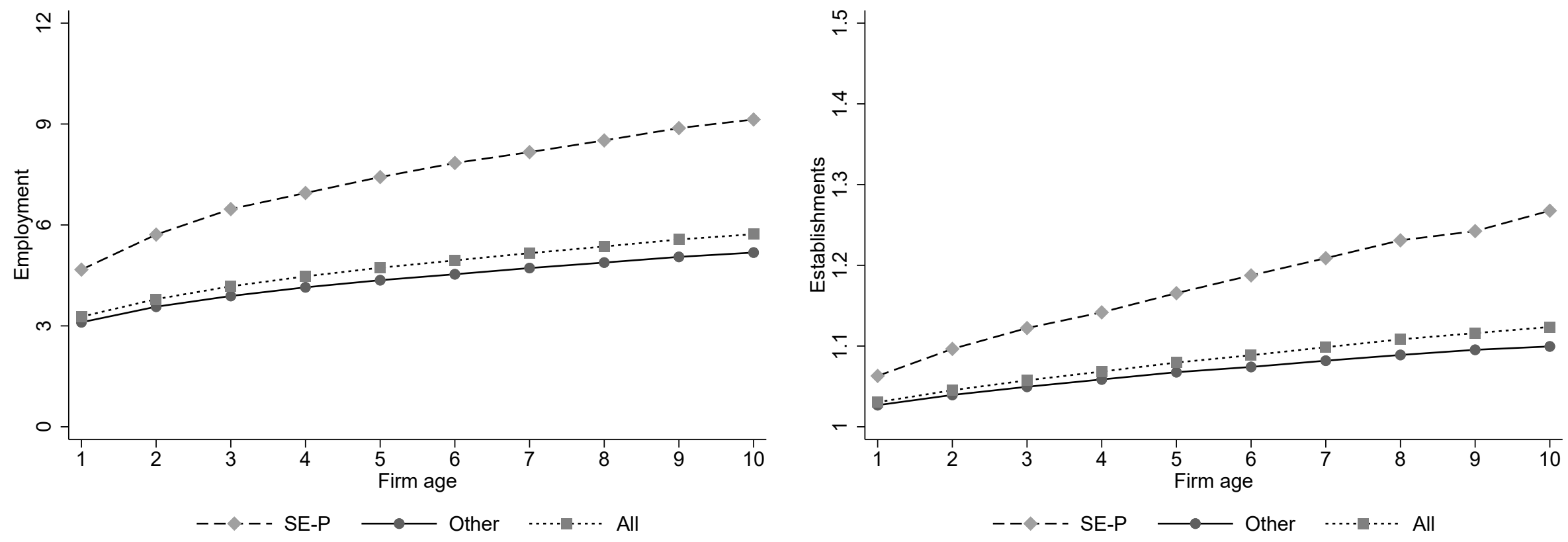

Notes: The figure shows the average size of single-establishment firms (left panel) and the average number of establishments per firm (right panel). It does so for serial entrepreneur (SE-P), other and all firms.

B.8 Margins of growth

In this Appendix, we investigate the margins of firm growth. In particular, we focus on when and how businesses tend to open more establishments ("extensive margin") or when and how they choose to employ more workers per establishment ("intensive margin").

Let us begin by investigating intensive margin growth. We do so first by focusing on single-establishment firms only, where intensive margin growth is the only option. The left panel of Figure A13 shows that single-establishment serial entrepreneur firms not only start larger but also grow faster compared to the other firms. In particular, employment growth is almost a third faster among SE-P firms (8.3% on average over the first ten years) compared to other firms (6.3%).[13] These patterns closely mimic those of all firms, suggesting that extensive margin growth is quantitatively not that important. Indeed, multi-establishment firms are quite rare in Portugal.

Specifically, only about 1.7% of other firms have multiple establishments at startup, while this fraction is about 3.5% for serial entrepreneur businesses. Nevertheless, conditional on having multiple establishments, serial entrepreneur businesses tend to open somewhat more establishments over the course of their life-cycle than other firms, see the right panel of Figure A13. Interestingly, both types of multi-establishment firms tend to enter with roughly the same average number of establishments (around

[13]In contrast, multiple-establishment regular businesses tend to expand their workforce *per establishment* at a somewhat faster pace (3.5% vs 3% among serial entrepreneur multi-establishment firms). However, it remains the case that serial entrepreneur multi-establishment firms enter larger. This holds true even per establishment.

2.7). However, by the time firms reach the age of 10 years, multi-establishment serial entrepreneur firms have 3.5 establishments on average, while other multi-establishment businesses operate about 20% few establishments – just under 3 on average.

Figure A14: Firm size distribution and its changes

(a) Firm size distribution (b) Size growth in firm size quartiles

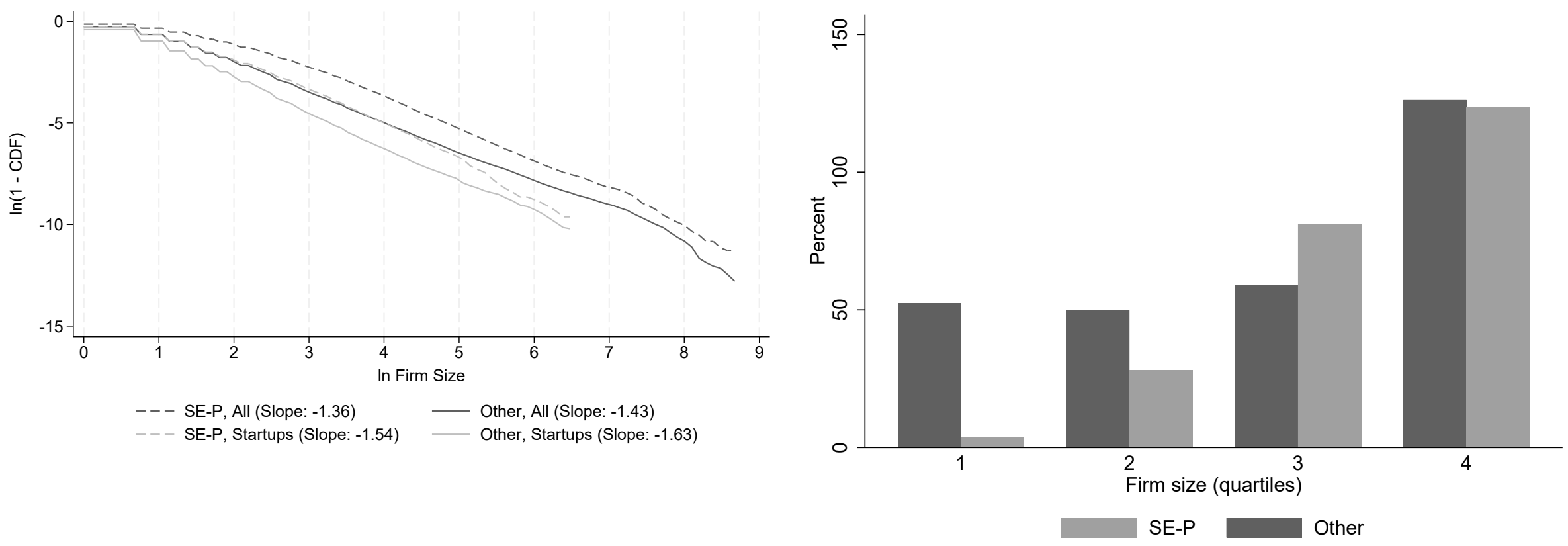

Notes: The figure shows the firm size distribution of serial entrepreneur and other firms (left panel) and their evolution by plotting the size growth (between startups and 10 year old firms) in the quartiles of the respective distributions (right panel). In the left panel, the horizontal axis displays log(employment), while the vertical axis shows log(1-CDF).

B.9 Implications for the Firm Size Distribution

In this Appendix, we report the implications of our findings for the firm-size distribution. Understanding the latter is of importance as it can inform about underlying frictions inhibiting firm growth (see for example Cabral and Mata, 2003; Hsieh and Klenow, 2014) as well as serving as a key target disciplining macroeconomic models (see for example Hopenhayn and Rogerson, 1993; Sterk ⓡ al., 2021; Jaimovich et al., 2023).

Firm size distributions overall. Let us now turn to investigating the implications of our previous findings for the firm size distribution. As is well known, Portugal (as well as other advanced economies) displays a size distribution which is skewed towards small firms (see for example Haltiwanger et al., 2013; Cabral and Mata, 2003). This pattern holds also within the groups of SE-P and other firms. However, the extent is much weaker for firms of serial entrepreneurs. We illustrate this in Figure A14 which displays the complementary cumulative distribution function in log-log scale, a figure typically used in the literature (see for example Axtell, 2001; Gabaix et al., 2016). The advantage of this particular type of figure is that it also efficiently demonstrate whether the data is consistent with Pareto's Law.

The left panel of Figure A14 shows the firm size distributions of SE-P and other firms. It does so separately for startups and all other and SE-P firms. First, note that the tails of the size distributions of both (all) other and SE-P firms can be approximated

by a straight line, indicating Pareto tails.[14] However, SE-P firms are clearly skewed towards larger businesses. Moreover, this occurs essentially across the entire firm size distribution suggesting that larger average sizes of SE-P firms are not because of ”only” a handful of very large firms. For example, while only 3% of other firms employ more than 20 workers, this fraction is 13% among SE-P businesses.

Firm size distributions over the life-cycle. The apparent differences in firm size between SE-P and other firms suggest different sources of – or impediments to – firm growth among the two types of businesses. One possibility is that SE-P firms are larger from the onset. The left Panel of of Figure A14 shows that size differences between the two groups of firms indeed exist already at startup. These results, therefore, suggest that SE-P businesses may enjoy stronger innate characteristics (“ex-ante heterogeneity”) or they may face more favorable initial conditions. We return to these points in the next section when formally investigating the underlying sources of the superior performance of SE-P firms. However, not all differences between the two groups of firms need to be accounted for by initial conditions.

An alternative possibility is that serial entrepreneur firms find it easier to grow, either because of superior growth potential or weaker impediments to growth (for example due to better access to external funding from their first businesses). The right panel of Figure A14 speaks to the growth potential of SE-P and other firms by visualizing how the respective firm size distributions fan out with firm age.

In particular, the panel displays the size growth in the respective quartiles of the size distributions between startup and age ten. For example, the gray bar in the fourth quartile indicates that the largest 25% of SE-P firms at age 10 are about 125% bigger than the largest 25% of SE-P startups. The figure suggests that while below median regular firms tend to grow faster, it is above median SE-P businesses which tend to expand at a faster pace compared to the other firms. Taken together, the figures suggests that SE-P firms both start larger and those in the upper tail of the size distribution tend to exhibit stronger growth potential compared to other businesses.

[14]See Kondo et al. (2021) for a discussion of the differences between estimating Pareto vs log-normal distributions (or their mixtures).

B.10 Life-Cycle Patterns of Gazelles

In this Appendix, we provide information on the life-cycle patterns of high-growth firms. In doing so, we distinguish between those owned by serial entrepreneurs (SE-P) and other business owners.

Figure A15 plots average size and exit profiles for the groups of SE-P and other gazelles. The shaded areas in the figure indicate the five-year window used to define gazelles. Recall, however, that we follow high-growth firms even after that age and even if their growth slows.

The right panel confirms results from our estimated risk premia – SE-P gazelles are considerably less likely to shut down.[15] Note that the survival bias inherent to the study of serial entrepreneurship is somewhat less severe in Figure A15. This is because our definition of high-growth firms requires they survive for at least 3 years. Importantly, this applies to *both* SE-P and other businesses.

The left panel shows how gazelles grow on average over their respective life-cycles. While both types of gazelles enter with essentially identical startup sizes, SE-P gazelles display stronger growth early on in their life-cycle. This opens up a gap in firm size which, from about the age of five, remains relatively stable. For both SE-P and other gazelles, growth slows after the age of five. This is not surprising, given that this is the cutoff age used in the definition of high-growth firms (i.e. businesses with sustained high growth in the first five years of their existence). Insofar as firm growth is partly driven by favorable – but transitory – shocks, average growth of gazelles will fade after the age of five as such "luck" runs out.

[15] Given the definition of gazelles, we report exit rates only from age 3. They mechanically display an increasing pattern as initially high-growth firms need to survive in order to be classified as high-growth.

Figure A15: High-growth firms: Size and exit profiles by age

(a) Firm size

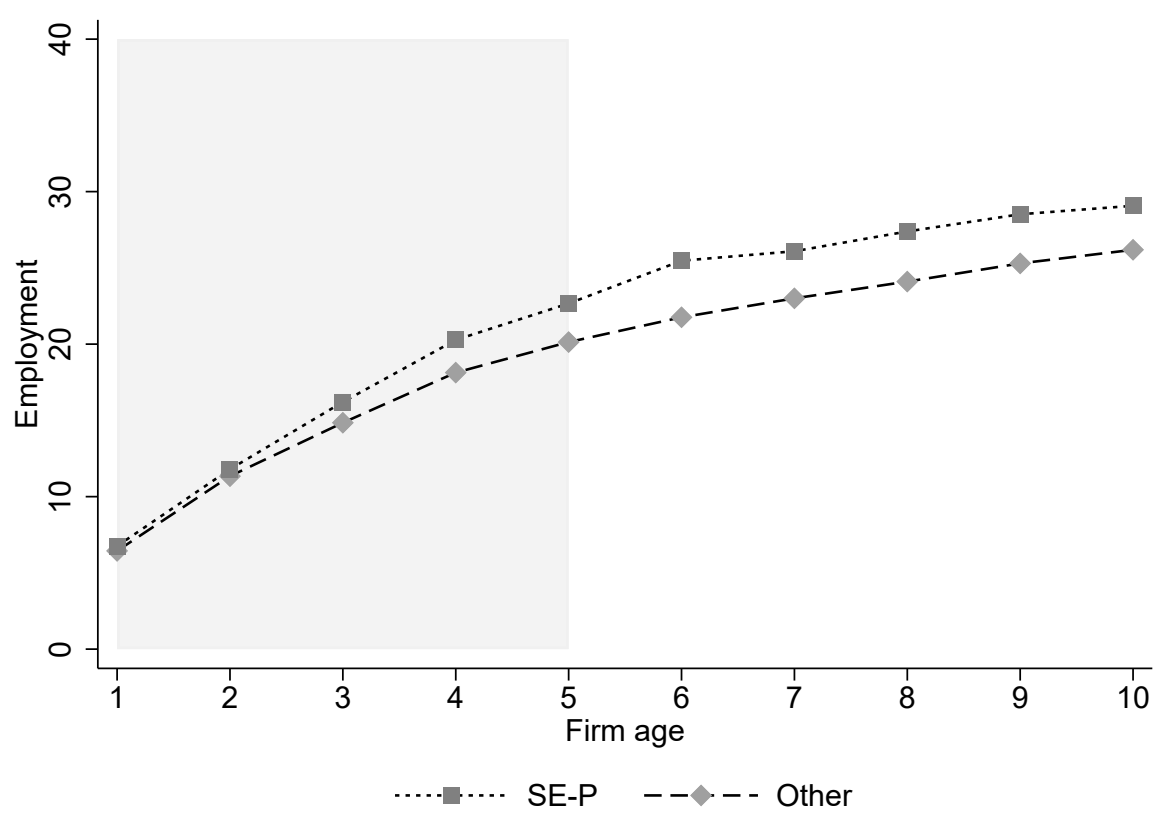

(b) Exit rates

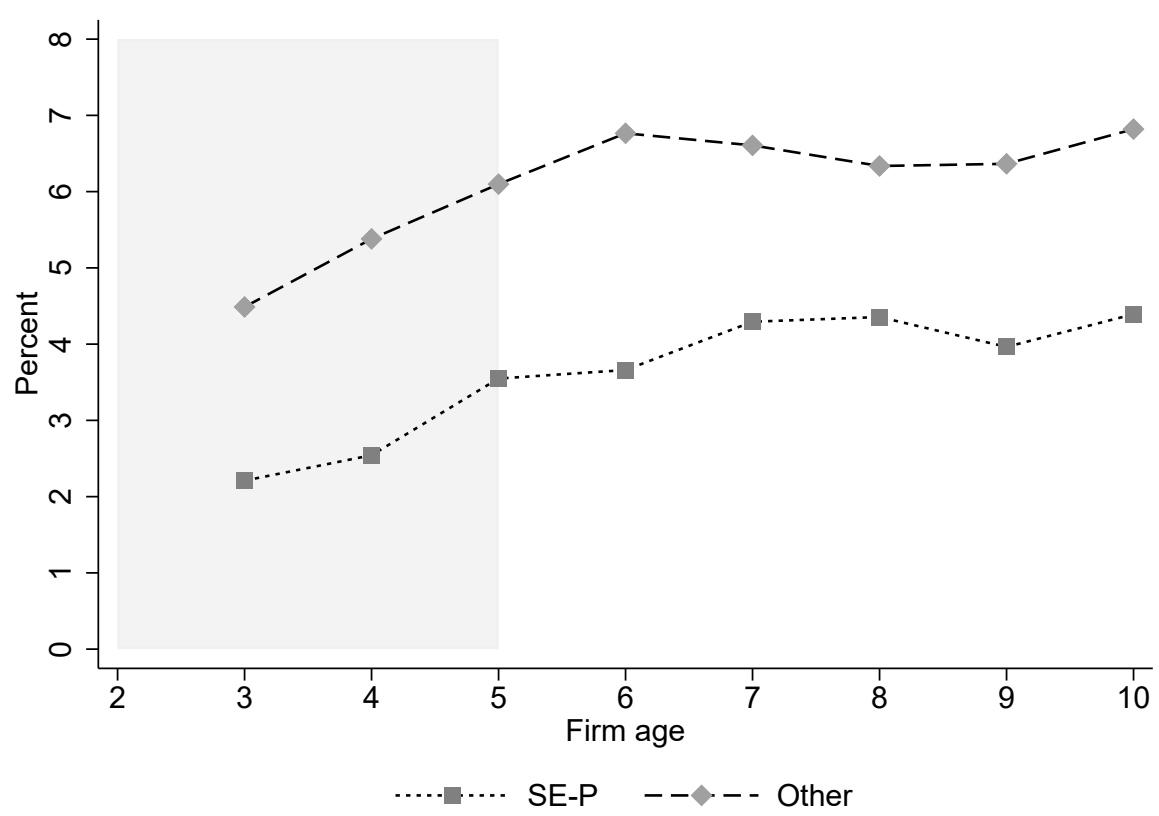

Notes: The left panel shows average firm size by age, while the right panel shows average exit rates by age. Both panels depict SE-P and other high-growth businesses. We define gazelles as businesses younger than five years which display an annual growth rate of at least 20% for at least 3 years and employ at least 10 workers at some point in their life-time (see European Commission, 2007). Shaded areas indicate the five year period used for defining high-growth firms.

B.11 Selection: Robustness Exercises

Section 5 in the main text investigates to what extent various observable characteristics can account for the estimated serial entrepreneur premia. In this Appendix, we provide various sensitivity checks of our baseline results.

Table A9: Summary statistics: Gelbach explanatory variables

	Entrepreneur char. only		Entrepreneur & other char.	
	Other	Serial	Other	Serial
**A: Entrepreneur characteristics**				
Age (years)	40.2	41.3	40.0	41.1
Female proportion (%)	30.9	23.7	30.7	23.6
Education (years)	9.2	10.6	10.4	11.4
Owner/manager (%)	93.2	98.0	94.5	98.7
Wages (log hourly rate)	0.232	0.482	0.313	0.537
Management experience (%)	19.5	52.1	21.6	51.9
**B: Workforce characteristics**				
Age (years)			39.7	39.7
Female proportion (%)			56.0	55.2
Education (years)			12.9	13.2
**C: Financial factors**				
Liquidity			2.15	2.18
Solvency			0.93	0.79
Cash ratio			0.93	0.68

Notes: The table reports average values for the explanatory variables considered in our SE premia regressions. All financial factors are ratios: Liquidity=liquid assets/total liabilities, solvency=total liabilities/total assets, cash ratio=cash and cash equivalents/current liabilities.

Explanatory variables. Table A9 provides average values for all the explanatory variables used in the our serial entrepreneur premia regressions. Recall that in the main text, entrepreneur characteristics and financial factors alone can explain up to 80% of the estimated SE premia. Within these, entrepreneur education, past wages and the solvency ratio are the most important factors (they alone can account for up to 2/3 of the estimated SE premia).

As can be seen from Table A9, serial entrepreneurs are more educated and command higher wages prior to starting a business. In particular, on average they have 1-1.4 years of extra education (depending on the sample being used) and earn about 25% more than other entrepreneurs just before starting their first business. In terms of solvency, serial entrepreneur firms enjoy a liability-to-asset ratio which is 15% lower compare to regular businesses.

Not Controlling for Entrepreneur Ability. One of the key drivers of the estimated SE premia are past wages of serial entrepreneurs. We view these as proxies for underlying

ability. In the main text, we use wages from the most recent year *prior to* moving into entrepreneurship. This, however, necessarily restricts our sample to only serial entrepreneurs for whom we observe the transition from paid work to entrepreneurship. Therefore, to check that our main results are not driven by this restricted sample, we re-estimate them while ignoring past wages.

Table A10 shows results for the case when we do not include wages as an explanatory variable. The results for the remaining explanatory factors remain relatively similar to those in the main text. In particular, entrepreneur characteristics alone explain between 14 and 34 percent of the estimated premia. Financial factors, and especially solvency, remain very important for exit and growth premia, accounting for 60 and 26, respectively. Finally, the age of workers also remains to play a role and explains between 15 and 41 percent of the exit and growth premia. Therefore, using the restricted sample in the main text – which allows us to control for entrepreneur ability – does not qualitatively affect our results.

Alternative Proxies for Entrepreneur Ability. Next, we re-estimate our results but instead of using wages from the most recent year prior to entrepreneurship, we consider average wages over (at most) three years prior to moving into entrepreneurship. The results are presented in Table A11. As can be seen, the estimated contributions change only very slightly. In fact, the contributions of entrepreneur characteristics to explaining the estimated SE premia increase somewhat when using this alternative measure.

First serial entrepreneur firms only. Financial factors were shown to play an important role in explaining the estimated SE premia. This is particularly true for solvency or, conversely, indebtedness. In this Appendix, we investigate the extent to which this is the case also for first serial entrepreneur (FSE) firms. The comparison between results from the main text and the ones in this Appendix then provide an indication of the extent to which subsequent serial entrepreneur firms can benefit from the (financial) success of their FSE predecessors.

Table A12 shows the results. All conclusions remain qualitatively the same as for our results in the main text. In particular, entrepreneur education, past wages, as well as solvency and to a lesser extent the age of workers in SE firms all explain sizable fractions of the estimated SE premia.

However, the contribution of financial factors is noticeably smaller when focusing on FSE firms only. Specifically, while for all firms, financial factors explain between 20 and 50 percent of the estimate exit and growth SE premia, these values are “only” 13 and 28 percent in the group of FSE firms. Therefore, it indeed seems to be the case that SSE firms partly benefit from the financial success of their FSE predecessors which allows them to operate with greater solvency ratios.

Table A10: Drivers of serial entrepreneur premia: No past wages

	Entrepreneur characteristics only				Entrepreneur & other characteristics			
	Size	Exit	Growth	Productivity	Size	Exit	Growth	Productivity
Unconditional SE premium, β^u	0.48	−1.90	1.60	0.31	0.45	−0.36	0.45	0.27
Conditional SE premium, β^c	0.41	−1.25	1.19	0.21	0.38	0.07	0.01	0.19
**A: Entrepreneur characteristics**								
Total contribution	0.06	−0.65	0.41	0.10	0.04	−0.16	0.15	0.06
Share of SE premium	14%	34%	26%	31%	10%	46%	32%	21%
Contributing factors:								
- *age*	−0.00	−0.04	−0.06	0.00	−0.00	−0.01	−0.07	0.00
- *gender*	0.00	−0.04	0.05	0.01	0.00	−0.02	0.05	0.01
- *education*	0.05	−0.26	0.37	0.07	0.04	−0.11	0.15	0.04
- *owner/manager*	0.01	−0.32	0.05	0.01	−0.00	−0.02	0.02	0.00
**B: Workforce characteristics**								
Total contribution					0.00	−0.05	0.18	0.01
Share of SE premium					1%	15%	41%	3%
Contributing factors:								
- *age*					0.01	−0.07	0.14	0.00
- *gender*					−0.00	0.00	−0.00	0.00
- *education*					−0.01	0.01	0.04	0.01
**C: Financial factors**								
Total contribution					0.02	−0.21	0.12	0.02
Share of SE premium					5%	60%	26%	7%
Contributing factors:								
- *liquidity*					0.00	−0.01	0.02	0.00
- *solvency*					0.01	−0.23	0.10	0.02
- *cash ratio*					0.01	0.03	0.00	0.00

Notes: The table reports results from estimating the SE premium. The first row reports "unconditional" serial entrepreneur premia, β^u, which ignore explanatory factors, $G_{i,t}$. The second row shows conditional serial entrepreneur premia, β^c, which control for $G_{i,t}$. The remainder of the table reports individual contributions of explanatory factors, grouped into "Entrepreneur characteristics" (Panel A), "Workforce quality" (Panel B) and "Financial factors" (Panel C). Each panel reports the "Total contribution" of the set of factors, "Share of SE premia" defined as (Total contribution)/β^u and the contributions of the underlying individual factors. The table reports results for a decomposition using only entrepreneur characteristics (first 4 columns) and all considered factors (last 4 columns).

Table A11: Drivers of serial entrepreneur premia: 3 years of past wages

	Entrepreneur characteristics only				Entrepreneur & other characteristics			
	Size	Exit	Growth	Productivity	Size	Exit	Growth	Productivity
Unconditional SE premium, β^u	0.46	−1.51	1.65	0.30	0.46	−0.40	0.52	0.28
Conditional SE premium, β^c	0.34	−0.80	1.03	0.16	0.34	0.04	0.00	0.15
**A: Entrepreneur characteristics**								
Total contribution	0.12	−0.71	0.61	0.14	0.10	−0.18	0.23	0.10
Share of SE premium	26%	47%	37%	46%	21%	44%	44%	35%
Contributing factors:								
- *age*	−0.01	−0.06	−0.09	0.00	−0.00	−0.02	−0.06	−0.00
- *gender*	0.00	−0.04	0.04	0.01	−0.00	−0.03	0.04	0.01
- *education*	0.02	−0.26	0.28	0.05	0.02	−0.18	0.11	0.03
- *owner/manager*	0.01	−0.21	0.03	0.00	−0.00	−0.02	0.03	0.00
- *past wages*	0.06	0.09	0.24	0.06	0.06	0.15	0.10	0.05
- *management experience*	0.04	−0.22	0.11	0.02	0.02	−0.08	0.00	0.01
**B: Workforce characteristics**								
Total contribution					0.00	−0.05	0.17	0.01
Share of SE premium					0%	14%	33%	3%
Contributing factors:								
- age					0.01	−0.06	0.14	0.00
- gender					0.00	−0.00	−0.00	0.00
- education					−0.01	0.01	0.03	0.01
**C: Financial factors**								
Total contribution					0.02	−0.21	0.11	0.02
Share of SE premium					5%	52%	22%	7%
Contributing factors:								
- liquidity					0.00	−0.00	0.01	0.00
- solvency					0.01	−0.23	0.10	0.01
- cash ratio					0.01	0.03	−0.00	0.00

Notes: The table reports results from estimating the SE premium. The first row reports "unconditional" serial entrepreneur premia, β^u, which ignore explanatory factors, $G_{i,t}$. The second row shows conditional serial entrepreneur premia, β^c, which control for $G_{i,t}$. The remainder of the table reports individual contributions of explanatory factors, grouped into "Entrepreneur characteristics" (Panel A), "Workforce quality" (Panel B) and "Financial factors" (Panel C). Each panel reports the "Total contribution" of the set of factors, "Share of SE premia" defined as (Total contribution)/β^u and the contributions of the underlying individual factors. The table reports results for a decomposition using only entrepreneur characteristics (first 4 columns) and all considered factors (last 4 columns).

Table A12: Drivers of serial entrepreneur premia: no SSE firms

	Entrepreneur characteristics only				Entrepreneur & other characteristics			
	Size	Exit	Growth	Productivity	Size	Exit	Growth	Productivity
Unconditional SE premium, β^u	0.34	−1.16	1.56	0.26	0.36	−0.55	0.69	0.25
Conditional SE premium, β^c	0.21	−0.64	0.75	0.14	0.23	−0.19	−0.08	0.13
**A: Entrepreneur characteristics**								
Total contribution	0.13	−0.51	0.81	0.12	0.10	−0.10	0.38	0.10
Share of SE premium	37%	44%	52%	47%	29%	18%	55%	38%
Contributing factors:								
- *age*	0.01	0.11	0.17	−0.00	0.01	0.05	0.12	0.00
- *gender*	0.00	−0.03	0.03	0.01	−0.00	−0.02	0.03	0.00
- *education*	0.02	−0.24	0.26	0.05	0.02	−0.17	0.10	0.03
- *owner/manager*	0.00	−0.17	0.02	0.00	−0.00	−0.02	0.02	0.00
- *past wages*	0.05	0.06	0.19	0.04	0.05	0.13	0.09	0.04
- *management experience*	0.04	−0.25	0.14	0.02	0.03	−0.08	0.02	0.02
**B: Workforce characteristics**								
Total contribution					0.01	−0.10	0.30	0.01
Share of SE premium					2%	18%	44%	3%
Contributing factors:								
- age					0.02	−0.12	0.26	0.00
- gender					−0.00	0.01	0.00	−0.00
- education					−0.01	0.01	0.04	0.01
**C: Financial factors**								
Total contribution					0.02	−0.16	0.09	0.01
Share of SE premium					5%	28%	13%	6%
Contributing factors:								
- liquidity					0.00	−0.01	0.01	0.00
- solvency					0.01	−0.17	0.08	0.01
- cash ratio					0.01	0.03	0.00	0.00

Notes: The table reports results from estimating the SE premium. The first row reports "unconditional" serial entrepreneur premia, β^u, which ignore explanatory factors, $G_{i,t}$. The second row shows conditional serial entrepreneur premia, β^c, which control for $G_{i,t}$. The remainder of the table reports individual contributions of explanatory factors, grouped into "Entrepreneur characteristics" (Panel A), "Workforce quality" (Panel B) and "Financial factors" (Panel C). Each panel reports the "Total contribution" of the set of factors, "Share of SE premia" defined as (Total contribution)$/\beta^u$ and the contributions of the underlying individual factors. The table reports results for a decomposition using only entrepreneur characteristics (first 4 columns) and all considered factors (last 4 columns).

References

Axtell, Robert (2001) "Zipf Distribution in U.S. Firm Sizes," *Science*, 293 (5536), 1818–1820.

Cabral, Luís M B and José Mata (2003) "On the Evolution of the Firm Size Distribution: Facts and Theory," *American Economic Review*, 93 (4), 1075–1090, http://dx.doi.org/10.1257/000282803769206205rm 10.1257/000282803769206205.

Caliendo, Lorenzo, Giordano Mion, Luca David Opromolla, and Esteban Rossi-Hansberg (2020) "Productivity and Organization in Portuguese Firms," *Journal of Political Economy*, 128 (11), 4211–4257, http://dx.doi.org/10.1086/710533rm 10.1086/710533.

Choi, Joonkyu, Nathan Goldschlag, John C Haltiwanger, and J. Daniel Kim (2021) "Early Joiners and Startup Performance," Working Paper 28417, National Bureau of Economic Research, http://dx.doi.org/10.3386/w28417rm 10.3386/w28417.

Davis, Steven, John Haltiwanger, and Scott Schuh (1996) *Job Creation and Destruction*, Cambridge, MA: The MIT Press.

European Commission (2007) "Eurostat-OECD Manual on Business Demography Statistics,"Technical Report KS-RA-07-010, https://ec.europa.eu/eurostat/web/products-manuals-and-guidelines/-/ks-ra-07-010.

Gabaix, Xavier, Jean-Michel Lasry, Pierre-Louis Lions, and Benjamin Moll (2016) "The Dynamics of Inequality," *Econometrica*, 84 (6), 2071–2111.

Haltiwanger, John, Ron S. Jarmin, Robert Kulick, and Javier Miranda (2016) *High Growth Young Firms: Contribution to Job, Output, and Productivity Growth*, 11–62: University of Chicago Press, http://www.nber.org/chapters/c13492.

Haltiwanger, John, Ron S. Jarmin, and Javier Miranda (2013) "Who Creates Jobs? Small versus Large versus Young," *The Review of Economics and Statistics*, 95 (2), 347–361, http://dx.doi.org/10.1162/REST$_{a0}$0288$rm10.1162/REST_{a0}0288$.

Hopenhayn, Hugo and Richard Rogerson (1993) "Job Turnover and Policy Evaluation: A General Equilibrium Analysis," *Journal of Political Economy*, 101 (5), 915–938.

Hsieh, Chang-Tai and Peter Klenow (2014) "The Life Cycle of Plats in India and Mexico," *Quarterly Journal of Economics*, 129 (3), 1035–1084.

Jaimovich, Nir, Stephen J Terry, and Nicolas Vincent (2023) "The Empirical Distribution of Firm Dynamics and Its Macro Implications," Working Paper 31337, National Bureau of Economic Research, http://dx.doi.org/10.3386/w31337rm 10.3386/w31337.

Jarmin, Ron and Javier Miranda (2002) "The Longitudinal Business Database," Center for Economic Studies Discussion Paper CES–WP–02–17.

Kondo, Illenin, Logan Lewis, and Andrea Stella (2021) "Heavy Tailed, but not Zipf: Firm and Establishment Size in the U.S.," Center for Economic Studies Working Paper 21-15.

Lima, Francisco and Pedro Telhado Pereira (2003) "Careers and wages within large firms: evidence from a matched employer-employee data set," *International Journal of Manpower*, 24 (7), 812–835, http://dx.doi.org/10.1108/01437720310502140rm 10.1108/01437720310502140.

Mata, José and Pedro Portugal (2004) "Patterns of entry, post-entry growth and survival: a comparison between domestic and foreign owned firms," *Small Business Economics*, 22 (3), 283–298, http://dx.doi.org/10.1023/B:SBEJ.0000022219.25772.carm 10.1023/B:SBEJ.0000022219.25772.ca.

Moscarini, Giuseppe and Fabien Postel-Vinay (2012) "The Contribution of Large and Small Employers to Job Creation in Times of High and Low Unemployment," *American Economic Review*, 102 (6), 2509–2539.

Queiró, Francisco (2022) "Entrepreneurial Human Capital and Firm Dynamics," *The Review of Economic Studies*, 89 (4), 2061–2100, http://dx.doi.org/10.1093/restud/rdab070rm 10.1093/restud/rdab070.

Shaw, Kathryn and Anders Sørensen (2019) "The Productivity Advantage of Serial Entrepreneurs," *ILR Review*, 72 (5), 1225–1261, http://dx.doi.org/10.1177/0019793919865501rm 10.1177/0019793919865501.

Sterk, Vincent ⓡ Petr Sedláček ⓡ Benjamin Pugsley (2021) "The Nature of Firm Growth," *American Economic Review*, 111 (2), 547–79, http://dx.doi.org/10.1257/aer.20190748rm 10.1257/aer.20190748.
